# Replicate Once Per Cell Cycle: Replication Control of Secondary Chromosomes

**DOI:** 10.3389/fmicb.2018.01833

**Published:** 2018-08-07

**Authors:** Florian Fournes, Marie-Eve Val, Ole Skovgaard, Didier Mazel

**Affiliations:** ^1^Unité Plasticité du Génome Bactérien, Département Génomes et Génétique, Institut Pasteur, Paris, France; ^2^UMR3525, Centre National de la Recherche Scientifique, Paris, France; ^3^Department of Science and Environment, Roskilde University, Roskilde, Denmark

**Keywords:** megaplasmids, chromids, *repABC*, iterons, replication initiation

## Abstract

Faithful vertical transmission of genetic information, especially of essential core genes, is a prerequisite for bacterial survival. Hence, replication of all the replicons is tightly controlled to ensure that all daughter cells get the same genome copy as their mother cell. Essential core genes are very often carried by the main chromosome. However they can occasionally be found on secondary chromosomes, recently renamed chromids. Chromids have evolved from non-essential megaplasmids, and further acquired essential core genes and a genomic signature closed to that of the main chromosome. All chromids carry a plasmidic replication origin, belonging so far to either the iterons or repABC type. Based on these differences, two categories of chromids have been distinguished. In this review, we focus on the replication initiation controls of these two types of chromids. We show that the sophisticated mechanisms controlling their replication evolved from their plasmid counterparts to allow a timely controlled replication, occurring once per cell cycle.

## Introduction

The genome of most of bacteria is carried by a single circular chromosome, which is replicated bi-directionally from a single origin in a highly controlled manner. Approximately 10% of the bacterial species have their genome divided in two, or more, large replicative DNA molecules, with a main chromosome, and one or several secondary replicons (second chromosomes and/or megaplasmids) (Harrison et al., 2010; Touchon and Rocha, 2016; diCenzo and Finan, 2017). Several evidences suggest that second chromosomes originate from plasmids that have been domesticated by their ancestral host to become *bona fide* chromosomes (Harrison et al., 2010). Plasmids could represent up to 30% of the bacterial genomes, and in some cases large plasmids were called megaplasmids. One of the founding events of plasmid or megaplasmid domestication involves the transfer of essential core genes from the main chromosome to the plasmid. Certainly because of their plasmid ancestry, all studied secondary chromosomes carry a plasmid-like replication system. In the alpha-proteobacteria *Rhodobacter sphaeroides* the secondary replicon carries a *repABC* replication system (Suwanto and Kaplan, 1989; Cevallos et al., 2008), while, all the species belonging to the *Vibrionaceae* family have a specific iteron plasmid-like replication system dedicated to their second chromosome (Okada et al., 2005). Nonetheless, mechanisms controlling the second chromosomes replication appear to be more sophisticated than that controlling plasmid replication. Combining essential core genes and plasmid-like replication origin, second chromosomes exhibit features of chromosomes and plasmids, and thus were named chromids. From now on, we will use this terminology for such replicons (Harrison et al., 2010).

Faithful transmission of genetic information from a mother cell to daughter cells requires cell cycle coordinated replication and segregation of the genetic material before cell division. Chromosomal replication has an elaborated control of when to start DNA replication (timing of initiation); an accurate replication-elongation stage and a termination that untangles the replicated chromosomes now ready for partitioning (Schekman et al., 1974; Reyes-Lamothe et al., 2012). Chromosomes differ from plasmids in part by their replication controls, both in terms of initiation process and by their integration to the cell cycle. Chromosome replication generally occurs once per cell cycle and responds to cell growth parameters. On the contrary, plasmids may replicate in a cell cycle independent manner and their replication can be initiated randomly during the cell cycle (Nordström and Dasgupta, 2006). That being said, this last affirmation has been for years subject to debate, as for example, the F and R1 model plasmids supposedly replicate at a particular time during the cell cycle (Zeuthen and Pato, 1971; Pritchard et al., 1975). Replication initiation of almost all replicons starts when the origin-specific replication initiator recognizes and binds motifs located in a well-defined origin region (Wegrzyn et al., 2016). With the exception of certain symbiotic species and few cyanobacteria, chromosomal DNA replication is initiated at a conserved replication origin, *oriC*, and is orchestrated by DnaA, the “universal” initiator of chromosomal replication in bacteria (Akman et al., 2002; Ohbayashi et al., 2016; Hansen and Atlung, 2018). Plasmid replication can be controlled either by the binding of an initiator to repeated sequences called iterons, or by a small antisense RNA (Chattoraj, 2000; Brantl, 2014; Gaimster and Summers, 2015). Chromids contain a replication origin related to the one of plasmids and thus have retained many of plasmid-like features. Megaplasmids and chromids seem both to share a more tightly controlled replication (Rasmussen et al., 2007; Frage et al., 2016). However, due to their large size, chromids probably necessitated additional mechanisms of initiation control, which permit a well-defined replication initiation mostly integrated to the cell cycle. Two major types of chromids are distinguished based on their replication mechanisms: iteron chromids and *repABC* chromids. The *repABC* chromids are exclusively found in the alphaproteobacteria and their replication is dependent on an operon composed of three genes: *repA*, *repB*, and *repC*. Even if, only RepC, the initiator, is essential for DNA replication, all three proteins RepA, RepB, and RepC are required to tightly control replication initiation (Pinto et al., 2012). The iteron chromids are found in the two other classes of proteobacteria (beta and gamma), and their replication origin is mainly composed of short repeated sequences, called iterons, localized near a gene encoding the replication initiator (Heidelberg et al., 2000; Du et al., 2016). *Vibrio cholerae* has served as the model for investigations of iteron chromids replication and its connection with the cell cycle.

Here we review and discuss the mechanisms controlling the replication initiation of these two types of chromids: iteron and *repABC*. We highlight the complex levels of control found in chromids, compared to those of their ancestral plasmids, which allow chromids to replicate once, and only once, per cell cycle. We also discuss the timing of replication initiation of the iteron and *repABC* chromids and their integration to the cell cycle.

## From Megaplasmids to Chromids

### Origin of Chromids

**FIGURE 1:**
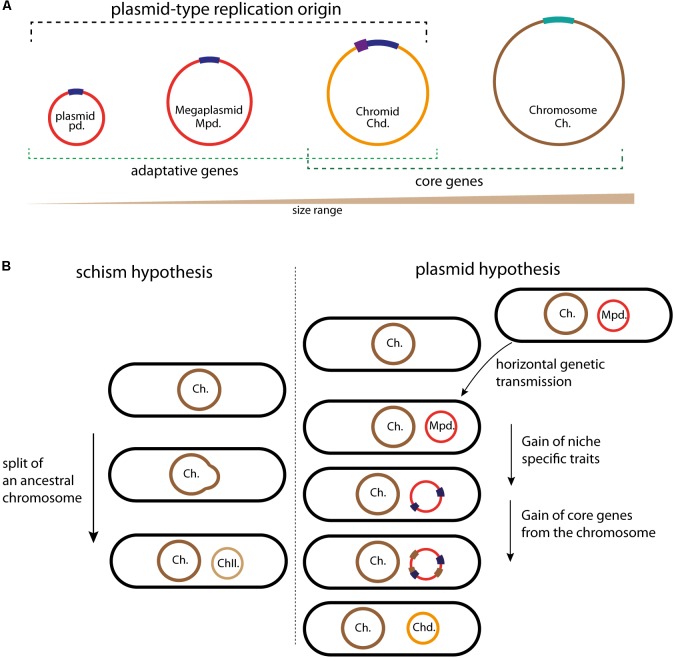
Schematic illustrating the different circular replicons found in bacterial genome and the chromids formation. **(A)** Classification of the bacterial replicons in function of their size. Plasmids, megaplasmids, and chromids carry a plasmid-type replication origin in dark blue; the additional regulatory sequences found in some chromids are represented in purple. The chromosome replication origin (*oriC*) is in light bleu. Adaptative genes are brought by plasmids and megaplasmids (red) but also by chromids (orange). Chromids and chromosomes (brown) carry core genes. **(B)** Schematic representation of the two schism and plasmid hypotheses, allowing to the formation of second chromosomes and chromids, respectively. Color code is the same as in **(A)**. For the schism hypothesis, the ancestral chromosome (brown) splits in two replicons, the main chromosome (Ch., in brown) and the second chromosome (ChII., in light brown). This second chromosome then acquires a plasmidic origin by fusion with mobile plasmid (red), leading to a chromid formation. For the plasmid hypothesis, the acquisition of a megaplasmid (red) by horizontal gene transfer is followed by the acquisition of genes (blue) that provide a growth benefit in the novel niche. The transfer of essential genes (brown) from the chromosome transforms the megaplasmid in chromid, now indispensable.

Bacterial genomes always include one chromosome and may also include plasmids. Plasmids provide beneficial accessory traits for the organism, for example, antibiotics resistance and anabolic pathways, but do not carry essential genes and thus are dispensable (**Figure 1A**). On the contrary, chromosomes harbor essential genes and are indispensable. This dogma changed, first, with the identification of linear chromosomes and plasmids (Hirochika and Sakaguchi, 1982; Baril et al., 1989), and in 1989, when Suwanto and Kaplin, using a pulsed-field gel electrophoresis, discovered a large second replicon in the alpha-proteobacteria *R. sphaeroides* (Suwanto and Kaplan, 1989). This replicon carrying essential genes was called “second chromosome”. The definition of second replicons as chromosome is mostly based on their essentiality in the bacteria growth and survival. In the 1990s other chromids were identified in *Agrobacterium tumefaciens*, *Brucella melitensis*, *Leptospira interrogans*, and in several *Vibrio* species (Allardet-Servent et al., 1993; Michaux et al., 1993; Zuerner et al., 1993; Trucksis et al., 1998; Yamaichi et al., 1999). In parallel, large replicons were discovered and called megaplasmids (**Figure 1A**) (Rosenberg et al., 1982). Compared to chromids, megaplasmids are non-essential, they encode their own replication and partition system, and carry adaptative genetic information such as the capacity for *Shigella flexneri* to invade the eukaryotic cells or, for the *Rhizobiaceae* to create a symbiosis with legumes (Buchrieser et al., 2000; Marchetti et al., 2010). The difference between plasmids and megaplasmids is currently based on the replicon size, and it will be of great benefit to establish if specific and functional characteristics discriminate plasmids and megaplasmids (**Figure 1A**). Chromids are normally larger than the accompanying plasmids and smaller than the associate chromosome. Comparative analysis of the relative synonymous codon usage of bacterial replicons demonstrates that individual replicons have distinct codon usage characteristics, and that chromids are much closer in codon usage to chromosomes than to plasmids (Harrison et al., 2010; Petersen et al., 2013). This observation implies that chromids have been acquired earlier than plasmids and have spent more time in the same cellular environment as the associated chromosome. Thus, codon usage analysis can be useful to chromids classification, as it was the case with the *Rhodobacteraceae* (alpha-proteobacteria) genomes analysis (Petersen et al., 2013; Frank et al., 2015a). In addition to that, three main criteria have been proposed to robustly distinguish chromids from chromosomes and plasmids or megaplasmids (**Figure 1A**) (Harrison et al., 2010). Replicons called chromids use a plasmid type maintenance and replication system, harbor a nucleotide composition close to that of the chromosome and carry essential core genes that are found on the chromosome of other species (Harrison et al., 2010). Prediction of the essentiality of core genes located within chromids is largely based on automated gene annotations. Experimental validations have in some cases shown that the predicted essential gene actually is dispensable (Cheng et al., 2007; Agnoli et al., 2012). For instance, in the case of the replicon pSymB of *Sinorhizobium meliloti* the *minCDE* genes were predicted to be essential, nonetheless, disruption of the *minE* gene is possible and only provokes a nitrogen fixation defect involved in symbiosis (Cheng et al., 2007). However, pSymB also carries core genes in unique copy, such as *engA* and *tRNA^arg^* and can still be considered as a chromid (diCenzo et al., 2013). Furthermore, chromids can be dispensable under smooth laboratory conditions, but must be required to bacteria survival in the harsh natural environment (Dziewit et al., 2014; Frank et al., 2015b; Soora et al., 2015). Thus, it was proposed to subdivide chromids into two types: “primary” and “secondary” chromids (Dziewit et al., 2014). Primary chromids are indispensable for host viability, while secondary chromids are considered as “facultatively” essential (Dziewit et al., 2014). However, many secondary replicons, such as megaplasmids carrying, for example, antibiotic resistance genes, which are essential for bacterial growth in presence of theses antibiotics and yet are not considered as chromids. Then, environment-specific beneficial or essential genes are insufficient to associate a replicon with the chromid term (diCenzo and Finan, 2017). Thus, even if this subdivision of chromids would be useful, we should be aware that it has to be carefully used.

The comparison of the available data helps us to determine the extent of megaplasmids and chromids relationship. Two main adaptive traits differentiate megaplasmids and chromids, leading to a stable and cell cycle integrated replicon: the acquisition of genomic signatures similar to those of cognate chromosomes (GC content and codon usage to limit physiological perturbation) and of essential genes. Two hypotheses have been proposed to explain the formation mechanism of an essential secondary replicon (Moreno, 1998; Egan et al., 2005; Prozorov, 2008; Harrison et al., 2010; diCenzo and Finan, 2017) (**Figure 1**). The first, called schism hypothesis, proposes that the formation of second essential replicon is the consequence of a split of an ancestral chromosome into two replicons: main and second chromosomes (**Figure 1B**). The second chromosome could then acquire the plasmid like replication system by fusion with a mobile plasmid, then becoming a chromid (Harrison et al., 2010; diCenzo and Finan, 2017). This was originally proposed to explain the formation of chromids found in *Brucella suis* and *R. sphaeroides*, but it seems to be able to explain solely rare chromids formation (Choudhary et al., 1997; Jumas-Bilak et al., 1998; diCenzo and Finan, 2017). Indeed, in bacteria, there is no evidence for the formation of chromids through the schism hypothesis. However, a recent study in the Archeon *Haloferax volcanii* describes the formation of a prokaryotic multipartite genome in agreement with the schism hypothesis. *H. volcanii* has a multipartite genome, consisting of a main chromosome, three secondary essential replicons and a plasmid, and its main chromosome has three origins, which are already well controlled (Norais et al., 2007; Hartman et al., 2010). In response to an *orc* gene deletion (*orc* encode the replication initiator Orc1), the multi-origin chromosome of *H. volcanii* split by homologous recombination into two elements, thus leading to the creation of a stable second chromosome (Ausiannikava et al., 2018). Contrary to the first hypothetical model, the second, called plasmid hypothesis, states that chromids evolved from megaplasmids (**Figure 1B**). This hypothesis implies that the coevolution of a megaplasmid with a chromosome will result in a transformation of the megaplasmid genomic signatures to that of the chromosome. This transformation is accompanied by the acquisition of essential genes (**Figure 1B**). This is supported by examples belonging to both the *repABC* and iterons chromids, which all carry a plasmid-like replication system and harbor a codon usage similar to that of the chromosome (Harrison et al., 2010; Pinto et al., 2012). Furthermore, the distribution of essential genes and the functional annotation onto the chromids are different compared to those of the chromosomes (Heidelberg et al., 2000; Goodner et al., 2001; Chao et al., 2013). As introduced above, these steps of evolution are the two main adaptive traits of a stable replicon. Strikingly, all observations gathered so far concluded that the plasmid hypothesis could explain the formation of all the studied chromids.

The acquisition of essential genes, prerequisite to the chromid formation, is driven by gene transfers from the chromosome to a megaplasmid (**Figure 1B**). Two possible mechanisms can explain the transfer of essential genes (diCenzo and Finan, 2017). First, inter-replicon genetic transfers could be catalyzed by homologous recombination, for example, by shared insertion sequences (IS), or IS using replicative transposition and resolution by recombination between different IS copies (Lesic et al., 2012). This transfer of genes leads to essential gene deletion from the chromosome. For instance, this is the case for the *engA* and the *tRNA^arg^* genes in the chromid pSymB, which resulted from the transfer of a 69Kb DNA fragment from the *S. meliloti* chromosome to the pSymB ancestor (diCenzo et al., 2013). On the other hand, the second mechanism takes into account the genetic redundancy due to inter-replicon gene duplication or to the acquisition of an orthologous gene by lateral genetic transfer. Several such examples of redundancy have been pointed in the genome sequences of *V. cholerae*, *R. sphaeroides* and *S. meliloti* (Heidelberg et al., 2000; Bavishi et al., 2010; diCenzo and Finan, 2015). For instance, massive inactivation experiments in *S. meliloti* chromosome has shown that more than 10% of the chromosomal genes have redundant functional copy on the megaplasmid pSymA or on the chromid pSymB, and this is a possible consequence of genes duplication (diCenzo and Finan, 2015).

### Where and Why Multipartite Genomes Appeared?

Bacterial genomes carried by more than one large replicon, thus containing megaplasmids and/or chromids, correspond to a divided or a multipartite genome. Increase in genome sequencing over the last years revealed that approximately 10% of the complete bacterial genomes are multipartite (Harrison et al., 2010; Touchon and Rocha, 2016; diCenzo and Finan, 2017). Multipartite genomes are found allover the bacterial kingdom but chromids are mainly found in proteobacteria, including the alpha, beta, and gamma proteobacteria (Harrison et al., 2010). Interestingly, megaplasmids are rarely conserved among genera, but are common in genera containing bacteria involved in symbiotic and pathogenic relationship. Furthermore, they carry genes specific to strains and species. In contrast, chromids are conserved among different genera and carry genus specific characters and genes (Harrison et al., 2010). For instance, pSymA is present only in few closely related *S. meliloti* species, and there is a high genes variation in individual strains (Cevallos et al., 2008; Guo et al., 2009). On the other hand, pSymB is supposed to be an old acquired replicon, sharing common ancestry with *Brucella* chromids, and pSymB chromids belonging to *S. meliloti* genomes show a high synteny between different isolates (Cevallos et al., 2008; Guo et al., 2009; Galardini et al., 2013). Thus, even if it could be difficult to differentiate chromids from megaplasmids with a systematic study of the genome, these observations may be key criteria to distinguish the two replicons. Besides the fact that chromids carry indispensable core genes, the advantages of multipartite genomes are not yet clearly established. Several hypotheses have been proposed. Multipartite genomes could allow bacteria to have a larger genome, and reduce the complexity of the circular replicons, which permit to correctly manage their heredity (*e.g.*, resolution of chromosome dimers) (Val et al., 2008, 2012). Indeed, the total genome size of the multipartite genomes are on average larger than the non-multipartite genomes, and the differences in genome sizes is correlated to the chromids size and not to the chromosomal size (diCenzo and Finan, 2017). In agreement with the previous hypothesis, the fast growing rhizobia contain a chromid contrary to the slow growing rhizobia (Yamaichi et al., 1999; Pastorino et al., 2003; MacLean et al., 2007). A second hypothesis is that chromids could permit the coordination and regulation of gene expression, contributing to the bacteria adaptation into novel niches. For instance, genes carried by *V. cholerae* chromid are differentially expressed *in vitro* and during the colon colonization. Indeed, during colon infection, *V. cholerae* induces a higher expression of chromid genes (Xu et al., 2003). These genes are involved in response to environmental stresses, allowing intra-intestinal growth and biofilm formation (Xu et al., 2003; Silva and Benitez, 2016).

The previous paragraphs highlighted the prevalence of chromids and their essentiality in the bacterial kingdom. The following sections will present what we know about their maintenance in the cell, focusing on the replication system of the iterons and *repABC* chromids.

## Iteron-Chromids and *Vibrio cholerae* Paradigm

The genome of *V. cholerae* is divided in two replicons of different sizes: the main chromosome (Chr1) of 3 Mbp and the chromid (Chr2) of 1 Mbp (Trucksis et al., 1998; Yamaichi et al., 1999; Heidelberg et al., 2000). Each replicon encodes a specific partition system, ParAB1 and ParAB2, which recognize different *parS* sites carried on their cognate replicons. Their replication is also differentially regulated (Duigou et al., 2006; Yamaichi et al., 2007). The replication origin of Chr1 is highly related to the chromosomal origin of *Escherichia coli*, and is controlled by the ubiquitous replication initiator DnaA (Duigou et al., 2006). The control of the replication by DnaA is elaborate, and involves, in addition to the regulation of the DnaA concentration in the cell, a balance of the binding affinity of DnaA to multiple sites within or outside the replication origin. The different levels of control of the DnaA replication process have been recently reviewed in (Hansen and Atlung, 2018). The *V. cholerae* main chromosome origin (*ori1*) contains DnaA binding sites, an IHF binding site and several GATC sites for methylation catalyzed by the DNA adenine methyl-transferase (Dam). Dam methylation is not essential to initiate the replication of Chr1, but SeqA, which recognize the hemi-methylated DNA, is required to restrict *ori1* initiation once per cell cycle (Demarre and Chattoraj, 2010). *ori1* can functionally replace the *E. coli*, *oriC*, and sustains chromosome replication (Koch et al., 2010). DnaA can bind ATP or ADP, but only ATP-DnaA can initiate the chromosomal replication initiation (Hase et al., 1998; Kawakami et al., 2005; Katayama et al., 2010; Hansen and Atlung, 2018). The regeneration of the ATP-DnaA, from the ADP-DnaA, is crucial for chromosome replication control. One of the mechanisms catalyzing this regeneration involves two intergenic regions called DARS1 and DARS2 (DnaA Reactivating Sequence) (Fujimitsu et al., 2009). DARS-like sequences are also found, with the same localization (between *uvrB* and *mutH*), in *V. cholerae* (Fujimitsu et al., 2009). All together, these observations suggest that *V. cholerae* Chr1 and *E. coli* chromosomes share many similar mechanisms to control their initiation.

This, however, does not exclude the involvement of *V. cholerae* species-specific elements to control the DnaA dependent replication. Indeed, the replication regulation of *Bacillus subtilis* and *Caulobacter crescentus*, two other model bacteria, which also use DnaA as initiator, involves additional and specific factors (Murray and Errington, 2008; Scholefield et al., 2012; Duan et al., 2016; Felletti et al., 2018). For example, Soj, an homolog of the partition protein ParA, controls the replication initiation during the *B. subtilis* vegetative growth (Ogura et al., 2003). Soj performs two opposite activities depending on its monomeric or dimeric state. Indeed, Soj monomers inhibit replication by preventing DnaA oligomerization (Murray and Errington, 2008; Scholefield et al., 2012). Conversely, Soj dimers, which require binding to ATP, activate replication by promoting DnaA oligomerization (Murray and Errington, 2008; Hansen and Atlung, 2018). *E. coli* has no *par* genes, but as mentioned above *V. cholerae* has one for each chromosome, and the *V. cholerae parB1* deletion induces Chr1 over-initiation; the same phenomenon is observed with an over-expression of ParA1, suggesting that ParA1 stimulates chromosome replication initiation as Soj does in *B. subtilis* (Kadoya et al., 2011).

### Players in the Replication of the *V. cholerae* Chromid: *ori2* and RctB

**FIGURE 2:**
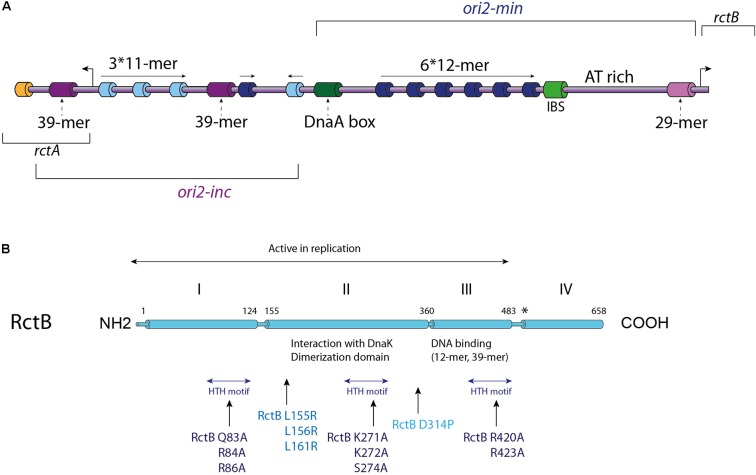
*V. cholerae* Chr2 replication initiation. **(A)** Linear Representation of the Chr2 origin (*ori2*), the two distinct parts of *ori2*: the replicative part (*ori2-min*) and the regulatory part (*ori2-inc*) are indicated. Each type of RctB binding site is represented with a different color: iterons (12-mers) in dark blue, 11-mers in light blue, 39-mers in purple and the 29-mer in light purple. A DnaA-box (dark green), an IHF binding site (IBS – light green) and a *parS2* site (orange) are represented, as well as the AT-rich region also called DUE (DNA unwinding element). The location of the *rctB* gene and the *rctA* ORF are indicated. **(B)** Representation of RctB primary structure. The active replicative form of RctB and its four domains are indicated, as well as the dimerization domain, the interaction domains with DnaK and the DNA interaction domains (12-mer/39-mer and the three HTH domains). Some important mutations are highlighted: mutations within the three HTH motifs, and mutations within the dimerization domain, for which the impacts are described in the text. Mutations in RctB L155R, L156R, and L161R are located in the DnaK/RctB interaction domain and impede the interaction of RctB with DnaK, which normally enhance RctB monomerization.

*Vibrio cholerae* chromid, Chr2, carries a different replication origin (*ori2*) compared to the origin of the main chromosome (**Figure 2A**). Initiation of the replication at *ori2* is catalyzed by a specific factor named RctB, which is highly conserved within the *Vibrionaceae* family. The ∼900 bp *ori2* has retained many of iteron-plasmid features for replication control. *Ori2* is organized into two functional domains: *ori2-min*, which supports the replication alone and an adjacent sequence, *ori2-inc*, which acts as a negative regulator of replication (**Figure 2A**). Both parts contain a variety of RctB binding sites, which are named based on their length: 11-mers, 12-mers, 29-mer, and 39-mers (**Figure 2A**). The iterons, 11-mers and 12-mers, are closely related, without any similarity with the 29-mer and 39-mers. The 29-mer corresponds to a truncated 39-mer, missing 10 nt in its center (Venkova-Canova et al., 2012). The *ori2-min* harbors an array of six 12-mers oriented in a head-to-tail manner with a regular spacing of 10 or 11 base pairs and each 12-mer contains a GATC Dam methylation site. As *ori1*, *ori2* also contains a DnaA binding site, though a single one, and an IHF binding site (IBS) (**Figure 2A**). Furthermore, *ori2* DnaA binding site is required for the Chr2 replication but DnaA is not limiting to control the timing of replication initiation, suggesting that it must have another function (Duigou et al., 2006). The exact implication of the DnaA binding site and of the IBS in Chr2 initiation is still unknown (Gerding et al., 2015; Schallopp et al., 2017). DnaA binding sites have been found in the replication origin of many plasmids (Lu et al., 1998; Wegrzyn et al., 2016), and two hypotheses have been proposed for the possible role of DnaA in plasmid replication. First, it has been suggested that DnaA could help the stabilization of the origin opening catalyzed by the plasmid replication initiators (Rep proteins), and second that DnaA was needed for the helicase loading. Thus, it is tempting to think that DnaA and IHF have conserved the same hypothetic regulatory functions for *V. cholerae* Chr2 replication initiation. Moreover, a recent study showed that DnaA negatively regulates the replication of a mini R1-1 plasmid (Yao et al., 2018). This observation suggests that DnaA, bound to *ori2*, could be also involved in a negative regulation of the *ori2* replication initiation, interacting with RctB. The remaining part of *ori2-min* contains an A-T rich region and a 29-mer RctB binding site overlapping the *rctB* promoter (**Figure 2A**). The regulatory *ori2-inc* part is mainly composed of one 39-mer and of a second 39-mer found at the outskirt, overlapping a transcribed but non-translated ORF *rctA*. 39-mers do not contain Dam methylation site. Four 11-mers containing GATC sites and one single 12-mer are also located in *ori2-inc* (Venkova-Canova and Chattoraj, 2011) (**Figure 2A**). All these sites are known to play a replication initiation regulatory role, which we will describe below.

RctB is a 658 amino acids protein consists of four domains and its sequence has no detectable homology with other replication initiator (Orlova et al., 2017) (**Figure 2B**). RctB, with a molecular mass of 75.3 kDa is larger than other chromosomal or plasmidic initiator proteins, suggesting that it performs additional functions compared to DnaA and Rep proteins. The first 500 residues, including domains I, II, and III, are sufficient to promote *ori2* replication initiation (Yamaichi et al., 2011; Jha et al., 2012; Koch et al., 2012) (**Figure 2B**). The domain IV is supposed to mediate protein-protein interaction, and thus play a regulatory role in the RctB oligomerization on the origin (Yamaichi et al., 2011; Koch et al., 2012; Orlova et al., 2017). Recent structural and biochemical studies of domains II and III showed that RctB adopts a head-to-head dimeric form in solution (Jha et al., 2017; Orlova et al., 2017) (**Figure 2B**). Interestingly, the structure of these two central domains exhibit significant similarities with plasmid-type Rep proteins, including π from the R6K plasmid and RepE from the F plasmid (Komori et al., 1999; Swan et al., 2006). Despite the fact that domains III and IV was predicted to be a dimerization interface (Jha et al., 2014), structure of the RctB dimer, restricted to domains II and III, shows that the interaction is mediated by the domain II. Furthermore, substitution of a proline within the beta strand closest to the dimer interface disrupts dimer formation and produces a monomeric mutant in the full length RctB (D314P; **Figure 2B**) (Orlova et al., 2017).

**FIGURE 3:**
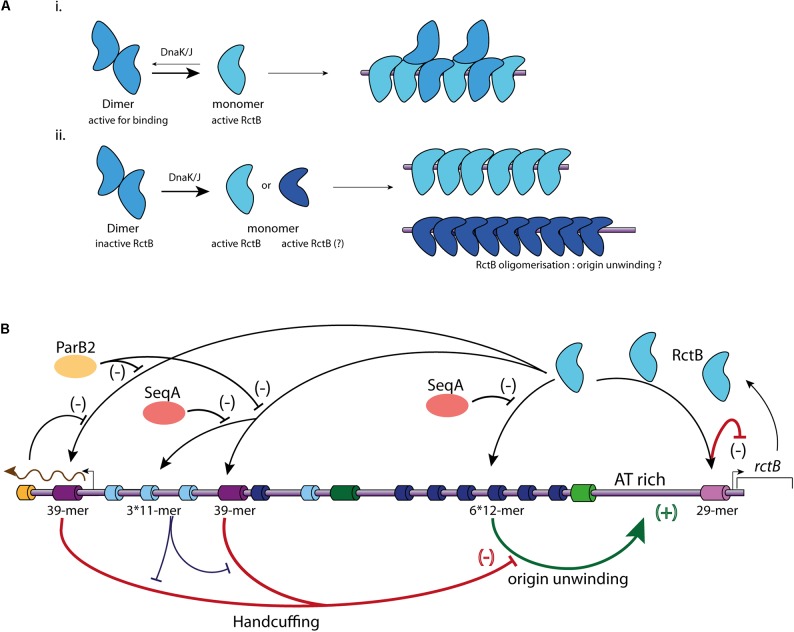
Main regulatory mechanisms controlling Chr2 replication initiation. **(A)** Representation of the two different models of RctB binding to the iterons. **(i)** RctB dimers, in blue, are transformed in monomers (light blue) by DnaK/J. Both dimers and monomers are able to binds to the iterons. **(ii)** RctB binding to the iterons is only possible under its monomeric form. The DnaK/J interaction with RctB not only causes its monomerization, but also its oligomerization (dark blue) onto the DNA containing iterons allowing to the origin unwinding. **(B)** Representation of the mechanisms involved in *ori2* replication initiation. RctB binding sites within the *ori2* are indicated and color codes are identical to those of the **(A)**. A black arrow illustrates RctB binding to its binding sites. A positive control is represented by a green arrow associated to (+), and a negative control is represented by flat end red arrow associated to (–). SeqA (orange) impedes the RctB binding to iterons, ParB2 (yellow) and *rctA* transcription (brown arrow) impede the RctB binding to 39-mers (bar black arrows). The handcuffing of the 39-mer with iterons within *ori2-inc* has a positive control on *ori2* replication initiation since it competes with the 39-mer handcuffing with *ori2-min* iterons (bar blue arrow).

As RctB is the *Vibrio* central player of chromid replication initiation, it should be able to take on different functions. The first of these is the recognition and binding to its target sites. The interaction between RctB and the 12-mer and 11-mer is dependent of the DNA methylation state, while its binding to the 39-mer and the 29-mer is methylation independent (Demarre and Chattoraj, 2010; Venkova-Canova et al., 2012). DNA/protein interaction experiments, using different RctB mutants, revealed that the domains interacting with the 12-mer and the 39-mer are spatially close and localized in the domain III (**Figure 2B**) (Jha et al., 2014). It was first proposed that RctB binds to the methylated 12-mer both as a monomer and a dimer (Jha et al., 2012) (**Figure 3A**). However, the head to head dimeric form of RctB is incompatible with the head to tail arrangement of 12-mer within *ori2-min* (Orlova et al., 2017) (**Figure 3A**). The crystal structure reveals that RctB contains more DNA binding surface than previously thought, with at least three helix-turn-helix (HTH) motifs identified, each one localized in a given domain (I, II, and III) (**Figure 2B**). Mutations in these three HTH reduce the RctB binding to all its target sites suggesting that all this three HTH are involved in DNA interactions. Furthermore, mutations in the three domains do not exhibit the same behavior regarding binding activity to the 11–12-mers and to the 29–39-mers. Indeed, all three domain I, II, and III, seem to be involved in the methylation dependent DNA binding (12-mer and 11-mer), while only domain II is involved in the methylation independent binding (29-mer and 39-mer) (Orlova et al., 2017).

In the iteron-plasmids mechanism of replication initiation, DnaK and DnaJ enhance initiator binding to the origin (Wickner et al., 1991). DnaK and DnaJ were first discovered as factors required for the bacteriophage lambda replication and later as enhancers for the replication of plasmids containing iterons within their origin (Friedman et al., 1984; Wickner et al., 1991). Plasmid initiators can dimerize, but in general bind to the origin only as monomers. DnaK/DnaJ system helps to monomerize plasmid initiator and promote the replication initiation. Based on structural data of the plasmid initiators RepA and RepE, it was proposed that monomerization is not sufficient to initiate the replication, and that monomers have to be remodeled, likely to catalyze origin unwinding (Díaz-López et al., 2003; Giraldo et al., 2003; Nakamura et al., 2007). In solution the RctB dimeric form is the most stable, this implies that monomerization of the protein has to be triggered to permit DNA binding (Jha et al., 2017; Orlova et al., 2017). RctB is remodeled from dimer to monomer by the chaperones DnaJ and DnaK *via* an interaction between DnaK and RctB domain II (**Figure 3A**) (*cf.* mutations L155R, L156R, and L161R; **Figure 2B**) (Jha et al., 2014, 2017). For Chr2 replication initiation, DnaK and DnaJ are strictly required to promote *ori2* replication initiation, and were shown to promote RctB binding to both activating and inhibiting sites (12-mers and 39-mers) (Jha et al., 2012). That being said, the elucidation of the precise characteristics of the RctB-DNA interaction needs further structural and biochemical studies, for example, to experimentally show the incapacity of RctB dimer to bind DNA. RctB mutants reducing the dimerization (*e.g.*, F311P) are still DnaKJ dependent to initiate the replication, suggesting that RctB monomers have to be remodeled to correctly work (Jha et al., 2017) (**Figure 3A**). Once bound to the *ori2-min* 12-mer, RctB has to oligomerize to open the adjacent A-T rich region (unwinding activity). The nature of this last process remains obscure. Thus, experimental data determining the role of DnaK and J, the identification of the RctB domain(s) involved in its oligomerization, as well as the precise role of A-T rich sequences needed to stabilize the opening of *ori2* are still missing.

### *V. cholerae* Chromid Controls of Replication Initiation

*Vibrio cholerae* Chr2 replicate once per cell cycle, pointing to a tight control through the balance between positive and negative effectors (Egan and Waldor, 2003; Egan et al., 2004; Venkova-Canova and Chattoraj, 2011; Baek and Chattoraj, 2014; Val et al., 2016). To summarize, RctB acts on two major types of sites, the 12-mer (iteron) to promote the replication initiation by unwinding the AT-rich region, and the 39-mer to inhibit it (**Figure 3B**). In *E. coli*, a plasmid carrying the entire *ori2* replicates at a copy number equal to that of the *E. coli* chromosome, and a plasmid carrying only *ori2-min* has a copy number increased by about 10 fold. Furthermore, the addition of the 39-mer to a plasmid containing *ori2-min* drastically reduced the plasmid copy number in the cell (Venkova-Canova and Chattoraj, 2011; Koch et al., 2012; Messerschmidt et al., 2015). The two main mechanisms of inhibition correspond to (1) the RctB titration and (2) the handcuffing between the 39-mer and the *ori2-min* 12-mer mediated by RctB (**Figure 3B**) (Venkova-Canova and Chattoraj, 2011). The inhibitory activity of the 39-mer is central, and the majority of the mechanisms that enhance replication initiation modulate the RctB/39-mer interactions (Pal et al., 2005; Venkova-Canova et al., 2006; Yamaichi et al., 2011).

The regulatory function of the iterons found in the *ori2-inc* region is dual. Indeed, they have a titration activity, similar to the 39-mer, but, additionally, they help to restrain the 39-mer inhibitory activity by enhancing the handcuffing inside the *ori2-inc* region, thus releasing the *ori2-min* 12-mers (Venkova-Canova and Chattoraj, 2011) (**Figure 3B**). Furthermore, the ParB2 protein, which binds Chr2 specific centromeres localized closer to the *ori2-inc*, serves as RctB competitor for the 39-mers binding by two mechanisms: (1) spreading from the *parS2* site closer to the leftmost 39-mer and (2) direct interaction with the central 39-mer (Yamaichi et al., 2011; Venkova-Canova et al., 2013) (**Figure 3B**). In addition, as the leftmost 39-mer is covered by the *rctA* transcript, this also interferes with the RctB binding at this site and thus impede its inhibitory activity (Venkova-Canova et al., 2006) (**Figure 3B**). These mechanisms controlling the 39-mer/RctB interactions release RctB from the inhibitor sites, first decreasing the titration phenomenon and second the handcuffing. Furthermore, as found for DnaA, the concentration of available RctB in the cell controls the Chr2 replication initiation. Thus, RctB gene expression is also tightly controlled. RctB auto-regulates its own expression through binding to the 29-mer located in the *rctB* promoter, where it plays a role of transcriptional repressor and exerts a negative feedback regulation (Pal et al., 2005; Egan et al., 2006) (**Figure 3B**). This 29-mer is also implicated in the *ori2* iterons handcuffing and is able to functionally replace the 39-mer (Venkova-Canova et al., 2012). In addition to this transcriptional regulation, the RctB concentration available to initiate the replication is also significantly controlled by its titration on various regulatory sites. As introduced above, the *ori2-inc* iterons together with the 39-mers and 29-mer can titrate RctB and reduce RctB binding to the *ori2-min* replicative iterons. Chromatin immunoprecipitation (Chip-chip) experiments have revealed that RctB also binds to a number of sites clustered within a 74 Kbp sequence on the Chr2 located 40 Kbp away from the *ori2* (Baek and Chattoraj, 2014). This 74 Kbp sequence contains six RctB binding sites: five iterons and one 39-mer like sequence, which also negatively regulate the *ori2* replication initiation. This locus titrate RctB and inhibit the *ori2* replication initiation, its activity and localisation suggest that it is comparable to the *E. coli datA* titration locus (Kitagawa et al., 1998; Kasho and Katayama, 2013).

The mechanisms of control also involve the methylation state of *ori2*, which prevents the replication restart during the same cell cycle (Demarre and Chattoraj, 2010). Contrary to the Chr1 origin, *ori1*, the Dam methylation of *ori2* is strictly required for its replication initiation (Demarre and Chattoraj, 2010; Val et al., 2014). Indeed, a *dam* mutant of *V. cholerae* can survive only when Chr1 and Chr2 are fused (Val et al., 2014). *ori2* has an overrepresentation of Dam methylation sites and is thus subjected to sequestration by SeqA (**Figure 3B**) (Demarre and Chattoraj, 2010). The SeqA sequestration prevents the immediate re-initiation of the replication, as in the case of Chr1, by temporally inhibiting the full-methylation of the DNA and initiator binding. Thus, the RctB binding to the iterons, which is dependent on the DNA methylation, is integrated to the cell cycle contrary, to its binding to the 39-mers and 29-mer. This methylation binding balance is involved in the cell cycle control of the Chr2 replication initiation.

### Integration of Iteron-Chromids Initiation Replication to the Cell Cycle

**FIGURE 4:**
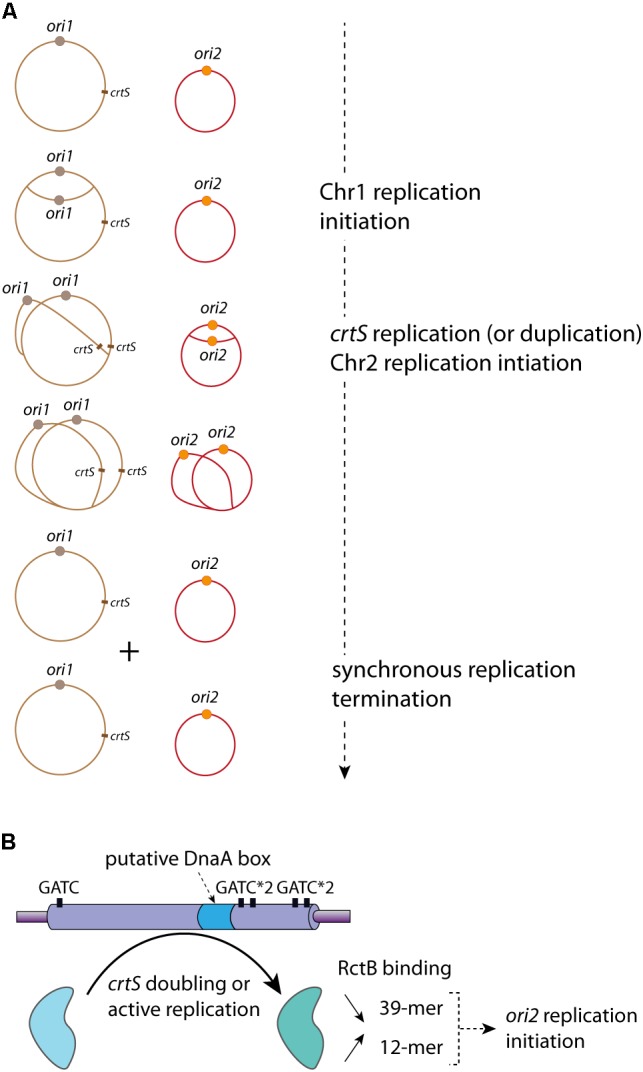
*crtS* controls the replication coordination of Chr1 and Chr2. **(A)** Coordinated Replication of Chr1 (in brown) and Chr2 (in red) in *V. cholerae*. The Chr1 origin *ori1* (brown circle) starts its replication initiation first. Once the *crtS* locus is replicated, Chr2 replication is triggered and occurs at o*ri2* (orange circle). Chr1 and Chr2 termination of replication is synchronous. The controlled replications of one Chr1 and one Chr2 of a mother cell lead to the formation of two Chr1 and two Chr2, which are equitably distributed in the daughter cells (not represented). **(B)** Representation of the *crtS* sequence composition, five GATC sites (black rectangle) and one putative DnaA-box (light blue) are indicated. The *crtS* chaperone activity, remodeling RctB to promote the *ori2* replication initiation, is represented by a black curved arrow (from the light blue to the light green form). The *in vivo* effects of *crtS* on the RctB binding to the iterons (12-mer) and the 39-mer are indicated: a black arrow oriented to the top represents the increasing interaction between RctB and 12-mer and a black arrow oriented to the bottom represents the decreasing interaction between RctB and the 39-mer.

In *V. cholerae*, Chr2 replication initiation is delayed compared to Chr1 replication initiation. Chr2 replication initiation starts when 2/3 of the replication period is completed. Besides, as Chr2 has a size equal to the 1/3 of Chr1, the replication termination of the two replicons is synchronous (Rasmussen et al., 2007) (**Figure 4A**). Marker frequency analysis (MFA) of a wide selection of Vibrios, with large variations in Chr1 and Chr2 sizes, suggests that there is a selective pressure for a termination synchrony, despite the fact that the control of Chr2 replication is at the initiation level (Kemter et al., 2018). Furthermore, in mutants where Chr2 finishes replicating earlier than Chr1, no impact on fitness was detected (Val et al., 2016). However, in these mutants the Chr2 terminus region (*ter2*) was shown to relocate earlier to mid-cell than in the *wt*, and remained localized at mid-cell until late in the cell cycle (Val et al., 2016). Despite early Chr2 replication termination, *ter2* retention at mid-cell suggests a secondary safeguard. How and why *ter2* segregation is delayed and results in re-synchronization with the Chr1 terminus region (*ter1*) is unknown. The mechanism coordinating the synchronous termination of the two replicons is driven by a locus found on the main chromosome. In *V. cholerae*, this locus, a short non-coding DNA sequence, is bound *in vivo* by RctB (Baek and Chattoraj, 2014). It is localized in the right replichore at around 800 Kbp downstream from *ori1*, and presents no homology with previously described RctB binding sites (*e.g.*, 12-mer and 39-mer) (Baek and Chattoraj, 2014). In *V. cholerae*, the deletion of this locus induces growth defects linked to cell filamentation and Chr2 loss (Val et al., 2016). Interestingly, moving the *V. cholerae crtS* to different location along the main chromosome led to a change of replication initiation timing of the Chr2 (Val et al., 2016). Replication of this Chr1 site triggers the replication of Chr2, which initiate after a short delay corresponding to the time needed for the replication of 200 Kbp. Thus, this checkpoint locus was named *crtS* for “chromosome 2 replication triggering site”. (Val et al., 2016) (**Figure 4A**). Besides, by employing chromosome conformation capture (3C) experiments, it has further been demonstrated that *ori2* and *crtS* are in a physical contact. These observations suggest that this *ori2* replication initiation regulatory mechanism could involve a structural interplay between Chr1 and Chr2 (Val et al., 2016). In *E. coli*, the presence of ectopic *V. cholerae* or *Vibrio nigripulchritudo crtS* increase the copy number of plasmids carrying different *ori2*, from *Vibrio tubiashi* or *Vibrio furnissi*. However, the copy number of plasmids containing the *ori2* of *Photobacterium profundum*, *Vibrio vulnificus*, or *Vibrio harveyi*, is not increased when *crtS* from other species (*e.g.*, *V. cholerae crtS* and *V. parahaemolyticus crtS*) are provided *in trans* (Kemter et al., 2018). These discrepancies could be due to the independence of the *P. profundum*, *V. vulnificus*, and *V. harveyi ori2* from *crtS* to regulate their replication, or to a species-specific mechanism. Thus, the *crtS* control activity is conserved, and *crtS* sites of divergent *Vibrio* species seem, to a certain extent, to be interchangeable for triggering the *ori2* replication initiation, showing a loose *crtS* species-specific activity (Kemter et al., 2018).

The alignment of different *crtS* sites shows a high sequence conservation among *Vibrionaceae*, including several GATC sites and a putative DnaA binding site (Baek and Chattoraj, 2014; Kemter et al., 2018) (**Figure 4B**). The RctB binding to *crtS* is hardly detected *in vitro* by DnaseI footprint experiments or by electrophoretic mobility shift assay (Baek and Chattoraj, 2014). It was proposed that, in *E. coli*, the *crtS* presence remodel RctB, decreasing its affinity for the 39-mer and conversely increasing it for the 12-mer (Baek and Chattoraj, 2014) (**Figure 4B**). This was drawn from *in vivo* data, but the *in vitro* experiments (electrophoretic mobility shift assay) did not allow obtaining clear results. Indeed, the authors observed only an *in vitro* decrease of RctB affinity to the 39-mer in presence of *crtS*, which could also reflect the competition between two types of RctB binding sites (Baek and Chattoraj, 2014). Thus, from these results it is difficult to differentiate a simple competition from an *in vitro crtS* remodeling activity. Moreover, in *E. coli* the presence of *crtS* makes DnaKJ dispensable for replication of *ori2* based plasmid (Baek and Chattoraj, 2014). This result, in addition to the effect of *crtS* on the RctB/DNA (12-mer and 39-mer) interactions, suggests a *crtS* DNA chaperone activity, which, by remodeling RctB, promotes Chr2 replication initiation. The *crtS* activity triggering *ori2* replication initiation is independent on methylation state of its GATC sites (de Lemos et al., in rev). However, the *crtS* form responsible for the DNA chaperone activity is still unknown. The passage of the replication fork across *crtS* would induce the formation of transient hemimethylated GATC sites, and the hemimethylated *crtS* may impact the RctB binding. Passage of the replication complex also generates single stranded DNA on the template of the lagging strand synthesis and could allow the formation of DNA hairpin. Thus, replication of *crtS* and the supposed DNA modifications it induces may be responsible for the *crtS* DNA chaperone activity. Nevertheless, the replication of *crtS* could simply lead to the duplication of the site, which could change the balance of free active RctB to catalyze the *ori2* opening. When already two copies of *crtS* were inserted on Chr1, Chr2 copy number was doubled suggesting that it is the presence of two *crtS* sites (after replication) that is important (Val et al., 2016). Indeed, a recent paper shows that the *crtS* duplication, without active replication, is sufficient to initiate *ori2* replication initiation (Ramachandran et al., 2018). However, it seems difficult to explain the *crtS* DNA chaperone activity solely from doubling its gene dosage. Further experimental data are needed to understand if either the active replication or the duplication of *crtS* is the signal controlling Chr2 replication initiation.

In conclusion, the molecular mechanisms by which the replication of *crtS* triggers the initiation of Chr2 through RctB are largely unknown. In *E. coli*, several mechanisms are responsible for the coordinated initiation of multiple origins (DnaA titration, regulatory inactivation of DnaA, origin sequestration and DnaA reactivation sequences) (Hansen and Atlung, 2018). All these mechanisms control the availability of the active form of DnaA in initiating replication from *oriC*. If the control of *ori2* initiation by *crtS* was performed only by controlling the availability of the RctB active form, we would expect a similar synchrony in the firing of multiple *ori2* and this would be observed by cells containing only 2^n^ *ori2* foci (*e.g.*, two or four). However, using cells with two chromosomal copies of *crtS*, the duplication of one *crtS* triggers the firing of only one *ori2* (Val et al., 2016). This suggests that Chr2 initiation firing may necessitate a contact between *crtS* and *ori2*. The contacts between *ori2* and Chr1, introduced above, may be caused by the simultaneous binding of RctB to *ori2* and *crtS* (Val et al., 2016). The most frequent contacts between *ori2* and Chr1 occur immediately downstream of *crtS*. A possible explanation is that, following the duplication of the *crtS* locus, the replication machineries of Chr1 and Chr2 are maintained in the vicinity of each other until the end of replication of the two chromosomes. Non-replicating cells (*i.e.*, stationary phase) lose the contacts observed between Chr1 and Chr2 replichores during exponential growth, suggesting that replication is indeed responsible for the contacts of the two chromosomes along their chromosomal arms. Overall, the 3C analysis of the *V. cholerae* chromosomes points to a direct interplay between 3D organization and replication regulation. How *trans* topological contacts would drive a functional interaction between the two chromosomes remains unknown.

## *Repabc* Chromids Replication Mechanisms and Controls

**FIGURE 5:**
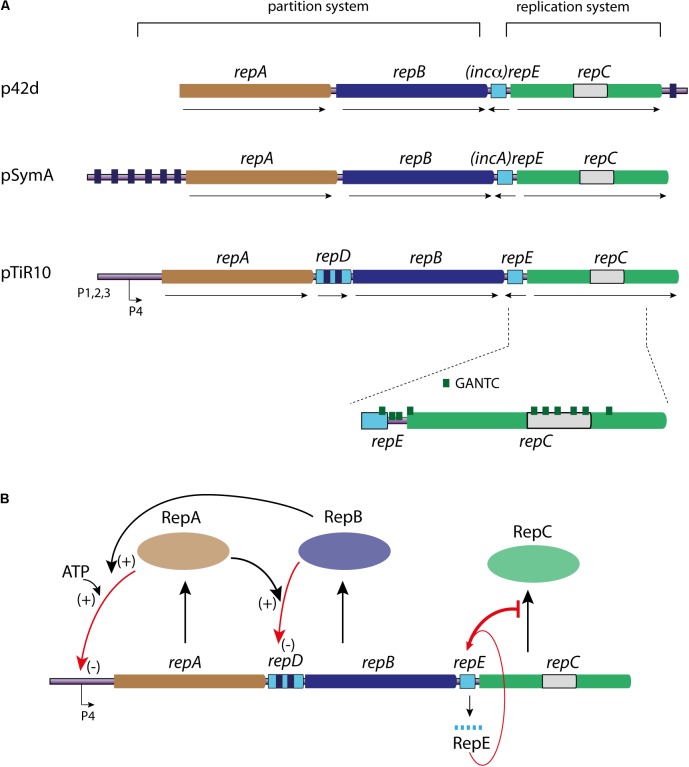
Linear representation of the genetic organization of *repABC* operons and RepC mechanisms of control. **(A)** The repABC operon is represented for three replicon models as indicated: p42d, pSymA, and pTiR10. The partition system is composed the genes *repA* (light brown) and *repB* (dark blue), and their cognate *parS* sites are represented by small dark blue boxes. The replication system is only composed of the *repC* gene (green), containing the RepC binding site (light gray). The counter transcribed (*repE*, *incA*, and *inc*α) is represented by a light blue rectangle. *repD* gene found in the pTiR10 *repABC* operon is represented by a light blue rectangle carrying two *parS* like sites (dark blue boxes). The number and localization of GANTC sites (green triangle), as the four promoters are only represented for pTiR10. For all the represented *repABC* operons the gene orientation corresponds to the black arrows. **(B)** Schematic representation of the replication initiation controls of the *repABC* chromids (pTiR10). Same color code as in **(A)**. RepA, RepB, and RepC proteins are represented by colored ovals. RepC production (black arrow) is controlled by RepE RNA (bar red arrow), which interacts with the *repB-repC* intergenic region. RepB binding to *repD* (red arrow) is enhanced by RepA (+), and controls *repB* and *repC* expression (–). RepA interaction with P4 promoter (red arrow) is enhanced by ATP and RepB (+), and this interaction controls the expression level of the whole operon (–). For further information, see text.

The genetic information of alpha-proteobacteria is commonly carried by a multipartite genome (Landeta et al., 2011; diCenzo and Finan, 2017). Whatever their nature, megaplasmids or chromids, the replication and segregation of those replicons involve, in most cases, three genes organized in operon: *repA, repB, and repC* (Galibert et al., 2001; Cevallos et al., 2008; García-de Los Santos et al., 2008; Petersen et al., 2013) (**Figure 5A**). The proteins encoded by the *repABC* operon are involved in two distinct mechanisms; RepC is essential for replication, and RepA and RepB are dispensable for replication but required for the partition. The *repA*, *repB*, and *repC* genes are expressed from promoters found upstream of *repA*. Most of our knowledge about the transcriptional regulation of the *repABC* operon comes from the *A. tumefaciens* megaplasmid pTiR10, where data show that *repABC* transcription is regulated by environmental cues (Ramírez-Romero et al., 2001; Pappas and Winans, 2003a,b). Indeed, the pTiR10 *repABC* operon contains four promoters (**Figure 5A**). The promoter P4 ensures the basal expression of the operon, but this promoter can be activated by the regulator VirG once phosphorylated by VirA, in response to plant pheromones (Cho and Winans, 2005). Furthermore, the pTiR10 four promoters are activated by the LuxR-family quorum sensing system (Pappas and Winans, 2003a).

### The Replication Initiator: RepC

RepC proteins are considered as the initiator protein of the *repABC* replicons and are found only in the alpha-proteobacteria (Palmer et al., 2000; Petersen et al., 2009). The *repC* gene alone is able to replicate a plasmid, showing that the origin is localized inside *repC* (Cevallos et al., 2008; Cervantes-Rivera et al., 2011; Pinto et al., 2011). At the structural level, the origin of the *repABC* replicons are lacking iterons and DnaA-boxes (Cervantes-Rivera et al., 2011; Pinto et al., 2012; Rajewska et al., 2012). The purified pTiR10 RepC binds to a 150 nt region containing an imperfect dyad near an AT-rich region. This sequence is localized in the middle of the *repC* coding sequence (**Figure 5A**) (Cervantes-Rivera et al., 2011; Pinto et al., 2011). RepC binds it cooperatively with a high specificity. Indeed, overexpression of RepC in *A. tumefaciens* induces an increase in plasmid copy number in *cis*, but does not change copy number of plasmids containing a parental origin in *trans*. Thus, RepC functions only in *cis*. The same phenomenon is observed for the RepC protein of the *R. elti* p42d replicon (Cervantes-Rivera et al., 2011; Pinto et al., 2011). RepC exhibits no homology with other replication initiators. Its predicted secondary structure suggests that RepC is divided in two domains: an amino-terminal (NTD) domain from residues 1 to 265 and a carboxy-terminal (CTD) domain from residues 298 to 439. The two NTD and CTD domains are connected by a linker peptide comprising 30 hydrophilic amino acids peptide (Pinto et al., 2011). The NTD domain of pTir10 RepC is essential for DNA binding but poorly contributes to the binding specificity, *a contrario*, the CTD domain is unable to bind the DNA alone but allows the discrimination between specific and non-specific binding (Pinto et al., 2011). Finally, in the case of the p42d RepC, the last 39 amino acids residues are shown to be involved in the incompatibility phenotype (Cervantes-Rivera et al., 2011). Inside the NTD domain, the region spanning residues 26–158 exhibits a structural similarity with the MarR family of transcription factors and is sufficient to bind the DNA. MarR binds DNA as a dimer, *via* a helix-turn helix (HTH) motif, suggesting that RepC can bind the DNA also as a dimer (Pinto et al., 2011). The supposed dimerization of RepC *via* its CTD domain has been proposed to play a role in the incompatibility between *repABC* replicons (del Solar et al., 1998; Cervantes-Rivera et al., 2011).

### Partition System and Replication Regulation

The control of replication initiation catalyzed by RepC is dependent of two major mechanisms, which both act on the *repC* expression level. These mechanisms involve the proteins RepA and RepB on one hand, and an antisense RNA on the other (**Figure 5**). RepA and RepB are members of the ParA and ParB families of partitioning proteins, and follow the same general mechanism of action (Williams and Thomas, 1992; Ramírez-Romero et al., 2000). The position and number of *parS* centromere-like site vary widely in the *repABC* replicons family (**Figure 5A**). These sites are essential for plasmid stability and are involved in the incompatibility mechanism between parental plasmids (MacLellan et al., 2006). Indeed, point mutations in the *parS* sites upstream the *repA2* of pSymA reduce the RepB binding and impede the incompatibility between pSymA parental plasmids. This incompatibility is presumably due to the competition between the two parental plasmids for the same partitioning system. RepA and RepB, together with *parS* sites, also participate to the negative transcriptional regulation of the operon, and thus act on the replication control of *repABC* replicons. Indeed, RepA binds to the *parS* sites and this binding may be enhanced by the presence of RepB and ATP. As an example, the RepA protein of pTiR10 auto-represses the P4 promoter, which is located within a 70 nt region protected against DnaseI digestion by RepA (Pappas and Winans, 2003b) (**Figure 5B**). Some bacteria belonging to the alpha-proteobacteria may have up to six *repABC* replicons; and the question of the RepA and RepB specific activity at their cognate sites and not at heterologous sites is still open. Two given RepA proteins share no more than 61% of identity and RepB proteins no more than 51%, this may be a key for avoiding cross interactions (incompatibility) (Cevallos et al., 2008; Castillo-Ramírez et al., 2009; Pinto et al., 2012). Thus, the high specific interactions between RepA, RepB and their cognate binding *parS* sites, together with proteins evolution and divergence, likely allow the coexistence of multiple *repABC* replicons in the same bacteria (Żebracki et al., 2015; Koper et al., 2016). In pTiR10-like replicons, a fourth transcribed and translated gene, *repD*, is located between *repA* and *repB* genes and contains two RepB binding sites (*parS*) (Chai and Winans, 2005b) (**Figures 5A,B**). It seems that the RepD protein is not involved in the replication and partition of pTiR10-like replicons (Chai and Winans, 2005b). The RepB binding to *repD* is enhanced by the presence of RepA. *repD* coding sequence is involved in the plasmid partitioning and negatively regulates *repB* and *repC* expression, adding another level of control to replication initiation (**Figure 5B**) (Chai and Winans, 2005b).

In addition to the negative regulation of the operon transcription by RepA and RepB, an antisense RNA also negatively regulates RepC (**Figures 5A,B**). This locus, located between *repB* and *repC*, encodes a 50 nucleotides antisense RNA (ctRNA) (Venkova-Canova et al., 2004; Chai and Winans, 2005a; MacLellan et al., 2005). This ctRNA includes a predicted stem-loop, which can act as a transcription terminator and form a complex with the *repABC* mRNA within the *repB-repC* intergenic region (Chai and Winans, 2005a) (**Figures 5A,B**). This RNA, known as RepE in pTir10, is conserved in most, if not all, replicons belonging to the *repABC* family (Cevallos et al., 2008). The RepE action model, proposed for the *A. tumefaciens* pTiR10 replicon by Chain and Winans, and supported by Cervantes-Rivera and collaborators for the *R. elti* p42d replicon, can be easily applicable to the other *repABC* replicons. In this model, the *repABC* mRNA can adopt two alternative secondary structures in the *repB-repC* intergenic region, depending to the presence or absence of RepE. In the absence of RepE, the intergenic region *repB-repC* is predicted to fold in a large stem-loop, leaving the *repC* Shine-Dalgarno sequence and its initiation codon single stranded, thus permitting the *repC* translation. In presence of RepE, its interaction with the target mRNA induces the re-folding of the sequence downstream of the interaction site, and creates two new stem-loops. One of the new stem-loops forms a Rho-independent termination site upstream of the *repC* ribosome-binding site leading to a premature termination (**Figure 5B**) (Chai and Winans, 2005a; Cervantes-Rivera et al., 2010). The *repB-repC* intergenic region, containing RepE, is also involved in the incompatibility between parental plasmids, and RepE was also named *incA* or *inc*α in plasmids pSymA and p42d, respectively (**Figure 5A**) (Ramírez-Romero et al., 2000; Soberón et al., 2004; MacLellan et al., 2005). Mutations reducing the RepE expression or remodeling its structure have been indeed shown to decrease the incompatibility (Chai and Winans, 2005a; Venkova-Canova et al., 2006; Pinto et al., 2012). All together, these mechanisms, *i.e*: the RepE ctRNA and the RepA/RepB negative regulation bring a fine tuning of the RepC expression level and thus control the replication initiation of the *repABC* replicons.

### Integration of *repABC*-Chromids Replication to the Cell Cycle

The replication and segregation of the alpha-proteobacteria multipartite genomes containing a *repABC* chromid is poorly documented. Nevertheless, the comparison of the data obtained for the bacteria *A. tumefaciens*, *S. meliloti*, and *B. abortus*, suggests the existence of a coordination mechanism for their two or three replicons (Kahng and Shapiro, 2003; Deghelt et al., 2014; Frage et al., 2016). The genome of *B. abortus* is divided in two replicons: the 2.1 Mbp chromosome and the 1.2 Mbp *repABC* chromid. The two replicons of *B. abortus* are oriented along the cell length axis, and the chromosome origin displays a bipolar orientation after its replication initiation, contrary to the chromid origin, which drift apart during the cell cycle and displays no sign of polar attachment (Deghelt et al., 2014). This last observation is similar to the results obtained for the *repABC* replicons of *A. tumefaciens* and *S. meliloti* (Kahng and Shapiro, 2003). Furthermore, the origin duplication of the *B. abortus* chromid occurs after the chromosome origin duplication and segregation of the chromid terminal region occurs before cell septation, while chromosome terminal region segregation is observed at the time of cell constriction. In the tripartite genome bacterium, *S. meliloti*, the partitioning of the three replicons (chromosomes, pSymA and pSymB) follows a highly conserved temporal order. The replication of the three replicons occurs once per cell cycle, and the segregation pattern is such that the chromosome segregates first, followed then by pSymA, and then by pSymB (Frage et al., 2016). Interestingly, the pSymA *repABC* region is sufficient to confer the spatiotemporal behavior of this replicon to a small plasmid. Besides, alterations of the DnaA activity, either positively or negatively, only impact the chromosome replication, and have no effect on the secondary replicons replication (Frage et al., 2016). Thus, it is likely that the strict timing of replication and segregation of *repABC* replicons only involve genetic components located within the repABC operon.

Finally, compared to the *V. cholerae* Chr2, there are no direct evidences of a subservient interplay between two replicons in the same cell in the alpha-proteobacteria, and thus no described mechanism. Nonetheless, the origin of replication and the promoters of the counter-transcribed *repE* gene of *repABC* chromids and mega-plasmids are rich in GANTC, which correspond to the Cell cycle-regulated Methylase (CcrM) methylation sites. In the alpha-proteobacteria *C. crescentus*, the A base of GANTC sites is methylated by CcrM (Marczynski and Shapiro, 2002; Wion and Casadesús, 2006). CcrM is functionally related to the *E. coli* methylase Dam, but there are important differences between them. Indeed, compared to Dam, which is active throughout the cell cycle, CcrM is synthesized and active only in predivisional cells. Unlike Dam, CcrM is not required for replication initiation or DNA mismatch repair (Gonzalez et al., 2014). However, CcrM overexpression results in abnormal chromosomes content per cell in *C. crescentus*. Thus, CcrM is essential for normal chromosomal replication. *C. crescentus* chromosome replicates once per cell cycle, and this seems to be controlled by the CcrM system (Stephens et al., 1996; Marczynski, 1999; Collier, 2012). CcrM is conserved across the alpha-proteobacteria and its orthologs has been studied in *S. meliloti*, *B. abortus*, and *A. tumefaciens* (Wright et al., 1997; Robertson et al., 2000; Kahng and Shapiro, 2001). Interestingly, with the notable exception of *C. crescentus*, the methylation of GANTC sites by CcrM seems to be essential in the other alpha-proteobacteria (Brilli et al., 2010; Fioravanti et al., 2013; Mohapatra et al., 2014). In the alpha-proteobacteria, a conserved master regulator, named CtrA, is involved in the control of the cell division and takes part in the spatio-temporal regulation of the replication initiation linked to the cell cycle (Wolański et al., 2014; Pini et al., 2015; Francis et al., 2017). CtrA is involved in the regulation of *ccrM* expression in both *C. crescentus* and *A. tumefaciens*, and it is likely the case in the other alpha-proteobacteria (Quon et al., 1996; Kahng and Shapiro, 2001). Therefore, the methylation state of the GANTC sites found in the *repABC* operon (*e.g.*, pTiR10) could be timely controlled, impacting the *repC* expression and *repE* transcription, and bringing a cell cycle integrated regulation of the *repABC* replication initiation. However, the *in vitro* binding of RepC to the origin is independent on the DNA methylation state (Pinto et al., 2011), but this does not exclude that other, yet unknown, replication factors might have a binding activity dependent on the GANTC methylation in the origin of *repABC* chromids.

## Conclusion and Remarks

In order to permit a faithful transmission of the genetic information, but also to avoid any problems due to polyploidy, chromids have to be replicated once and only once per cell cycle. In this review, we gave a short overview of chromid domestication history, and further focused our analysis on their replication and how they became integrated in the bacterial cell cycle. Most of our knowledge on chromid replication initiation comes from the *repABC* and iteron models, where controls mainly occur at the initiation step. Both types of chromid present multi-scale mechanisms to timely manage the replication initiation of the replicon, which first involves the recognition of the replication origin by the initiator protein (RepC/RctB). These controls are mostly centered on the initiator proteins both at the gene expression level and through the regulation of their specific activities. This first step is already controlled by diverse and numerous mechanism. Thus, iterons and *repABC* chromids seem to correspond to two different evolutionary ways of achieving a tight replication initiation control.

One of the mechanisms to avoid over-replication of iteron chromids is dependent on the Dam/SeqA couple. There is no SeqA homolog in the alpha-proteobacteria, but a yet unknown protein could play an analogous function of sequestration (Pinto et al., 2012). Besides, usually all the large replicons found in the alpha-proteobacteria carry a *repABC* operon, while iterons origins are only found in small plasmids in these bacteria (Szymanik et al., 2006). These observations suggest that iteron chromids, which are SeqA dependent, could not allow the tight replication initiation control of the alpha-proteobacteria megaplasmids and chromids.

After the initiator/origin interaction, the following steps, which correspond to the unwinding of the AT-rich region and to the recruitment of the replisome proteins, might also be regulated, but this has not been studied yet. In the case of the iterons plasmids, the recruitment and loading of the helicase DnaB involves a direct interaction of the helicase with DnaA and/or the plasmid initiator (Zhong et al., 2003; Wegrzyn et al., 2016). The interaction between RctB and DnaB has to be shown, as well as the DnaA binding to *ori2*, and its involvement in the DnaB loading. On the contrary, the *repABC* chromid origins do not contain DnaA boxes and thus it is tempting to think that RepC proteins interact directly with the helicase.

An important feature distinguishing the replication control of the two types of chromid is based on the existence (or not) of controls driven by other replicons. Indeed, the *repABC* multi-scale controls seem to be strictly intra-molecular, meaning that all the necessary sequences are carried by the replicon and located within the *repABC* operon (Frage et al., 2016). The results obtained with *B. abortus* and *A. tumefaciens* chromids reveal that these chromids initiate their replication once per cell cycle and after the chromosome (Kahng and Shapiro, 2003; Deghelt et al., 2014). This raises the question of how the *repABC* chromids can be replicated in synchrony with the main chromosome. In contrast, replication control of *Vibrio* iteron chromids involves an inter-molecular interaction (Val et al., 2016). The recent discovery of *crtS* and of the physical contacts between Chr1 and Chr2 reveals a unique checkpoint control of replication in bacteria (Baek and Chattoraj, 2014; Val et al., 2016). The determinants of this contact between Chr1 and Chr2 still have to be identified. Contacts between *crtS* and *ori2* may alter RctB binding and handcuffing activity, or other unknown process involved in Chr2 replication initiation. This new checkpoint implies a transfer of information between the two replicons, which apparently take a time equivalent to the replication of 200 Kbp (Val et al., 2016). This temporal delay corresponds to the time necessary to deliver the message of the *crtS* replication to the *ori2*, allowing to the remodeling of RctB activities and to the recruitment of the replisome, but the precise events and players involved in it, have yet to be determined.

At the moment, the reasons for the requirement of a replication delay for secondary replicons remains unknown. In *V. cholerae*, initiation of replication of Chr2 is delayed such that replication termination of Chr1 and Chr2 occurs at the same time. This could facilitate the coordination of the final steps of segregation before cell division. The location of *crtS* is highly conserved within the *Vibrio*. The *crtS* position may have been selected throughout evolution by the constraint imposed by this activation delay. The importance of multiple chromosomes to coordinate their replication and the importance for Chr1 and Chr2 to finish replicating at the same time remains in the realm of conjecture.

## Author Contributions

FF wrote the manuscript. All authors discussed and corrected the manuscript and approved it for publication.

## Conflict of Interest Statement

The authors declare that the research was conducted in the absence of any commercial or financial relationships that could be construed as a potential conflict of interest.

## References

[B1] Agnoli K. Schwager S. Uehlinger S. Vergunst A. Viteri D. F. Nguyen D. T. (2012). Exposing the third chromosome of *Burkholderia cepacia* complex strains as a virulence plasmid. *Mol. Microbiol.* 83 362–378. 10.1111/j.1365-2958.2011.07937.x 22171913

[B2] Akman L. Yamashita A. Watanabe H. Oshima K. Shiba T. Hattori M. (2002). Genome sequence of the endocellular obligate symbiont of tsetse flies, *Wigglesworthia glossinidia*. *Nat. Genet.* 32 402–407. 10.1038/ng986 12219091

[B3] Allardet-Servent A. Michaux-Charachon S. Jumas-Bilak E. Karayan L. Ramuz M. (1993). Presence of one linear and one circular chromosome in the *Agrobacterium tumefaciens* C58 genome. *J. Bacteriol.* 175 7869–7874. 10.1128/jb.175.24.7869-7874.1993 8253676 PMC206964

[B4] Ausiannikava D. Mitchell L. Marriott H. Smith V. Hawkins M. Makarova K. S. (2018). Evolution of genome architecture in Archaea: spontaneous generation of a new chromosome in *Haloferax volcanii*. *Mol. Biol. Evol.* 10.1093/molbev/msy075 [Epub ahead of print]. 29668953 PMC6063281

[B5] Baek J. H. Chattoraj D. K. (2014). Chromosome I controls chromosome II replication in *Vibrio cholerae*. *PLoS Genet.* 10:e1004184. 10.1371/journal.pgen.1004184 24586205 PMC3937223

[B6] Baril C. Richaud C. Baranton G. Saint Girons I. S. (1989). Linear chromosome of *Borrelia burgdorferi*. *Res. Microbiol.* 140 507–516. 10.1016/0923-2508(89)90083-1 2696058

[B7] Bavishi A. Lin L. Schroeder K. Peters A. Cho H. Choudhary M. (2010). The prevalence of gene duplications and their ancient origin in *Rhodobacter sphaeroides* 2.4.1. *BMC Microbiol.* 10:331. 10.1186/1471-2180-10-331 21192830 PMC3024229

[B8] Brantl S. (2014). Plasmid replication control by antisense RNAs. *Microbiol. Spectr.* 2:LAS-0001-2013. 10.1128/microbiolspec.PLAS-0001-2013 26104196

[B9] Brilli M. Fondi M. Fani R. Mengoni A. Ferri L. Bazzicalupo M. (2010). The diversity and evolution of cell cycle regulation in alpha-proteobacteria: a comparative genomic analysis. *BMC Syst. Biol.* 4:52. 10.1186/1752-0509-4-52 20426835 PMC2877005

[B10] Buchrieser C. Glaser P. Rusniok C. Nedjari H. D’Hauteville H. Kunst F. (2000). The virulence plasmid pWR100 and the repertoire of proteins secreted by the type III secretion apparatus of *Shigella flexneri*. *Mol. Microbiol.* 38 760–771. 10.1046/j.1365-2958.2000.02179.x 11115111

[B11] Castillo-Ramírez S. Vázquez-Castellanos J. F. González V. Cevallos M. A. (2009). Horizontal gene transfer and diverse functional constrains within a common replication-partitioning system in *Alphaproteobacteria*: the *repABC* operon. *BMC Genomics* 10:536. 10.1186/1471-2164-10-536 19919719 PMC2783167

[B12] Cervantes-Rivera R. Pedraza-López F. Pérez-Segura G. Cevallos M. A. (2011). The replication origin of a repABC plasmid. *BMC Microbiol.* 11:158. 10.1186/1471-2180-11-158 21718544 PMC3155836

[B13] Cervantes-Rivera R. Romero-López C. Berzal-Herranz A. Cevallos M. A. (2010). Analysis of the mechanism of action of the antisense RNA that controls the replication of the repABC plasmid p42d. *J. Bacteriol.* 192 3268–3278. 10.1128/JB.00118-10 20435728 PMC2897686

[B14] Cevallos M. A. Cervantes-Rivera R. Gutiérrez-Ríos R. M. (2008). The repABC plasmid family. *Plasmid* 60 19–37. 10.1016/j.plasmid.2008.03.001 18433868

[B15] Chai Y. Winans S. C. (2005a). A small antisense RNA downregulates expression of an essential replicase protein of an *Agrobacterium tumefaciens* Ti plasmid. *Mol. Microbiol.* 56 1574–1585. 10.1111/j.1365-2958.2005.04636.x 15916607

[B16] Chai Y. Winans S. C. (2005b). RepB protein of an *Agrobacterium tumefaciens* Ti plasmid binds to two adjacent sites between repA and repB for plasmid partitioning and autorepression. *Mol. Microbiol.* 58 1114–1129. 10.1111/j.1365-2958.2005.04886.x 16262794

[B17] Chao M. C. Pritchard J. R. Zhang Y. J. Rubin E. J. Livny J. Davis B. M. (2013). High-resolution definition of the *Vibrio cholerae* essential gene set with hidden Markov model-based analyses of transposon-insertion sequencing data. *Nucleic Acids Res.* 41 9033–9048. 10.1093/nar/gkt654 23901011 PMC3799429

[B18] Chattoraj D. K. (2000). Control of plasmid DNA replication by iterons: no longer paradoxical. *Mol. Microbiol.* 37 467–476. 10.1046/j.1365-2958.2000.01986.x 10931340

[B19] Cheng J. Sibley C. D. Zaheer R. Finan T. M. (2007). A *Sinorhizobium meliloti minE* mutant has an altered morphology and exhibits defects in legume symbiosis. *Microbiology* 153 375–387. 10.1099/mic.0.2006/001362-0 17259609

[B20] Cho H. Winans S. C. (2005). VirA and VirG activate the Ti plasmid repABC operon, elevating plasmid copy number in response to wound-released chemical signals. *Proc. Natl. Acad. Sci. U.S.A.* 102 14843–14848. 10.1073/pnas.0503458102 16195384 PMC1253548

[B21] Choudhary M. Mackenzie C. Nereng K. Sodergren E. Weinstock G. M. Kaplan S. (1997). Low-resolution sequencing of *Rhodobacter sphaeroides* 2.4.1T: chromosome II is a true chromosome. *Microbiology* 143(Pt 10), 3085–3099. 10.1099/00221287-143-10-3085 9353914

[B22] Collier J. (2012). Regulation of chromosomal replication in *Caulobacter crescentus*. *Plasmid* 67 76–87. 10.1016/j.plasmid.2011.12.007 22227374

[B23] Deghelt M. Mullier C. Sternon J.-F. Francis N. Laloux G. Dotreppe D. (2014). G1-arrested newborn cells are the predominant infectious form of the pathogen *Brucella abortus*. *Nat. Commun.* 5:4366. 10.1038/ncomms5366 25006695 PMC4104442

[B24] del Solar G. Giraldo R. Ruiz-Echevarría M. J. Espinosa M. Díaz-Orejas R. (1998). Replication and control of circular bacterial plasmids. *Microbiol. Mol. Biol. Rev.* 62 434–464. 9618448 10.1128/mmbr.62.2.434-464.1998 PMC98921

[B25] Demarre G. Chattoraj D. K. (2010). DNA adenine methylation is required to replicate both *Vibrio cholerae* chromosomes once per cell cycle. *PLoS Genet.* 6:e1000939. 10.1371/journal.pgen.1000939 20463886 PMC2865523

[B26] Díaz-López T. Lages-Gonzalo M. Serrano-López A. Alfonso C. Rivas G. Díaz-Orejas R. (2003). Structural changes in RepA, a plasmid replication initiator, upon binding to origin DNA. *J. Biol. Chem.* 278 18606–18616. 10.1074/jbc.M212024200 12637554

[B27] diCenzo G. Milunovic B. Cheng J. Finan T. M. (2013). The tRNA^arg^ gene and engA are essential genes on the 1.7-Mb pSymB megaplasmid of *Sinorhizobium meliloti* and were translocated together from the chromosome in an ancestral strain. *J. Bacteriol.* 195 202–212. 10.1128/JB.01758-12 23123907 PMC3553834

[B28] diCenzo G. C. Finan T. M. (2015). Genetic redundancy is prevalent within the 6.7 Mb *Sinorhizobium meliloti* genome. *Mol. Genet. Genomics* 290 1345–1356. 10.1007/s00438-015-0998-6 25638282

[B29] diCenzo G. C. Finan T. M. (2017). The divided bacterial genome: structure, function, and evolution. *Microbiol. Mol. Biol. Rev.* 81 e00019-17. 10.1128/MMBR.00019-17 28794225 PMC5584315

[B30] Du W.-L. Dubarry N. Passot F. M. Kamgoué A. Murray H. Lane D. (2016). Orderly replication and segregation of the four replicons of *Burkholderia cenocepacia* J2315. *PLoS Genet.* 12:e1006172. 10.1371/journal.pgen.1006172 27428258 PMC4948915

[B31] Duan Y. Huey J. D. Herman J. K. (2016). The DnaA inhibitor SirA acts in the same pathway as Soj (ParA) to facilitate oriC segregation during *Bacillus subtilis* sporulation. *Mol. Microbiol.* 102 530–544. 10.1111/mmi.13477 27489185

[B32] Duigou S. Knudsen K. G. Skovgaard O. Egan E. S. Løbner-Olesen A. Waldor M. K. (2006). Independent control of replication initiation of the two *Vibrio cholerae* chromosomes by DnaA and RctB. *J. Bacteriol.* 188 6419–6424. 10.1128/JB.00565-06 16923911 PMC1595377

[B33] Dziewit L. Czarnecki J. Wibberg D. Radlinska M. Mrozek P. Szymczak M. (2014). Architecture and functions of a multipartite genome of the methylotrophic bacterium *Paracoccus aminophilus* JCM 7686, containing primary and secondary chromids. *BMC Genomics* 15:124. 10.1186/1471-2164-15-124 24517536 PMC3925955

[B34] Egan E. S. Duigou S. Waldor M. K. (2006). Autorepression of RctB, an initiator of *Vibrio cholerae* chromosome II replication. *J. Bacteriol.* 188 789–793. 10.1128/JB.188.2.789-793.2006 16385068 PMC1347293

[B35] Egan E. S. Fogel M. A. Waldor M. K. (2005). Divided genomes: negotiating the cell cycle in prokaryotes with multiple chromosomes. *Mol. Microbiol.* 56 1129–1138. 10.1111/j.1365-2958.2005.04622.x 15882408

[B36] Egan E. S. Løbner-Olesen A. Waldor M. K. (2004). Synchronous replication initiation of the two *Vibrio cholerae* chromosomes. *Curr. Biol.* 14 R501–R502. 10.1016/j.cub.2004.06.036 15242627

[B37] Egan E. S. Waldor M. K. (2003). Distinct replication requirements for the two *Vibrio cholerae* chromosomes. *Cell* 114 521–530. 10.1016/S0092-8674(03)00611-1 12941279

[B38] Felletti M. Omnus D. J. Jonas K. (2018). Regulation of the replication initiator DnaA in *Caulobacter crescentus*. *Biochim. Biophys. Acta* 10.1016/j.bbagrm.2018.01.004 [Epub ahead of print]. 29382570

[B39] Fioravanti A. Fumeaux C. Mohapatra S. S. Bompard C. Brilli M. Frandi A. (2013). DNA binding of the cell cycle transcriptional regulator GcrA depends on N6-adenosine methylation in *Caulobacter crescentus* and other *Alphaproteobacteria*. *PLoS Genet.* 9:e1003541. 10.1371/journal.pgen.1003541 23737758 PMC3667746

[B40] Frage B. Döhlemann J. Robledo M. Lucena D. Sobetzko P. Graumann P. L. (2016). Spatiotemporal choreography of chromosome and megaplasmids in the *Sinorhizobium meliloti* cell cycle. *Mol. Microbiol.* 100 808–823. 10.1111/mmi.13351 26853523

[B41] Francis N. Poncin K. Fioravanti A. Vassen V. Willemart K. Ong T. A. P. (2017). CtrA controls cell division and outer membrane composition of the pathogen *Brucella abortus*. *Mol. Microbiol.* 103 780–797. 10.1111/mmi.13589 27893179

[B42] Frank O. Göker M. Pradella S. Petersen J. (2015a). Ocean’s Twelve: flagellar and biofilm chromids in the multipartite genome of *Marinovum algicola* DG898 exemplify functional compartmentalization. *Environ. Microbiol.* 17 4019–4034. 10.1111/1462-2920.12947 26079637

[B43] Frank O. Michael V. Päuker O. Boedeker C. Jogler C. Rohde M. (2015b). Plasmid curing and the loss of grip–the 65-kb replicon of *Phaeobacter inhibens* DSM 17395 is required for biofilm formation, motility and the colonization of marine algae. *Syst. Appl. Microbiol.* 38 120–127. 10.1016/j.syapm.2014.12.001 25595869

[B44] Friedman D. I. Olson E. R. Georgopoulos C. Tilly K. Herskowitz I. Banuett F. (1984). Interactions of bacteriophage and host macromolecules in the growth of bacteriophage lambda. *Microbiol. Rev.* 48 299–325. 6240590 10.1128/mr.48.4.299-325.1984 PMC373221

[B45] Fujimitsu K. Senriuchi T. Katayama T. (2009). Specific genomic sequences of *E. coli* promote replicational initiation by directly reactivating ADP-DnaA. *Genes Dev.* 23 1221–1233. 10.1101/gad.1775809 19401329 PMC2685538

[B46] Gaimster H. Summers D. (2015). Plasmids in the driving seat: the regulatory RNA Rcd gives plasmid ColE1 control over division and growth of its *E. coli* host. *Plasmid* 78 59–64. 10.1016/j.plasmid.2014.11.002 25446541 PMC4393325

[B47] Galardini M. Pini F. Bazzicalupo M. Biondi E. G. Mengoni A. (2013). Replicon-dependent bacterial genome evolution: the case of *Sinorhizobium meliloti*. *Genome Biol. Evol.* 5 542–558. 10.1093/gbe/evt027 23431003 PMC3622305

[B48] Galibert F. Finan T. M. Long S. R. Puhler A. Abola P. Ampe F. (2001). The composite genome of the legume symbiont *Sinorhizobium meliloti*. *Science* 293 668–672. 10.1126/science.1060966 11474104

[B49] García-de Los Santos A. López E. Cubillas C. A. Noel K. D. Brom S. (2008). Requirement of a plasmid-encoded catalase for survival of *Rhizobium etli* CFN42 in a polyphenol-rich environment. *Appl. Environ. Microbiol.* 74 2398–2403. 10.1128/AEM.02457-07 18310436 PMC2293148

[B50] Gerding M. A. Chao M. C. Davis B. M. Waldor M. K. (2015). Molecular dissection of the essential features of the origin of replication of the second *Vibrio cholerae* chromosome. *mBio* 6:e00973. 10.1128/mBio.00973-15 26220967 PMC4551981

[B51] Giraldo R. Fernández-Tornero C. Evans P. R. Díaz-Orejas R. Romero A. (2003). A conformational switch between transcriptional repression and replication initiation in the RepA dimerization domain. *Nat. Struct. Biol.* 10 565–571. 10.1038/nsb937 12766757

[B52] Gonzalez D. Kozdon J. B. McAdams H. H. Shapiro L. Collier J. (2014). The functions of DNA methylation by CcrM in *Caulobacter crescentus*: a global approach. *Nucleic Acids Res.* 42 3720–3735. 10.1093/nar/gkt1352 24398711 PMC3973325

[B53] Goodner B. Hinkle G. Gattung S. Miller N. Blanchard M. Qurollo B. (2001). Genome sequence of the plant pathogen and biotechnology agent *Agrobacterium tumefaciens* C58. *Science* 294 2323–2328. 10.1126/science.1066803 11743194

[B54] Guo H. Sun S. Eardly B. Finan T. Xu J. (2009). Genome variation in the symbiotic nitrogen-fixing bacterium *Sinorhizobium meliloti*. *Genome* 52 862–875. 10.1139/g09-060 19935910

[B55] Hansen F. G. Atlung T. (2018). The DnaA tale. *Front. Microbiol.* 9:319. 10.3389/fmicb.2018.00319 29541066 PMC5835720

[B56] Harrison P. W. Lower R. P. J. Kim N. K. D. Young J. P. W. (2010). Introducing the bacterial “chromid”: not a chromosome, not a plasmid. *Trends Microbiol.* 18 141–148. 10.1016/j.tim.2009.12.010 20080407

[B57] Hartman A. L. Norais C. Badger J. H. Delmas S. Haldenby S. Madupu R. (2010). The complete genome sequence of *Haloferax volcanii* DS2, a model archaeon. *PLoS One* 5:e9605. 10.1371/journal.pone.0009605 20333302 PMC2841640

[B58] Hase M. Yoshimi T. Ishikawa Y. Ohba A. Guo L. Mima S. (1998). Site-directed mutational analysis for the membrane binding of DnaA protein. Identification of amino acids involved in the functional interaction between DnaA protein and acidic phospholipids. *J. Biol. Chem.* 273 28651–28656. 10.1074/jbc.273.44.28651 9786858

[B59] Heidelberg J. F. Eisen J. A. Nelson W. C. Clayton R. A. Gwinn M. L. Dodson R. J. (2000). DNA sequence of both chromosomes of the cholera pathogen *Vibrio cholerae*. *Nature* 406 477–483. 10.1038/35020000 10952301 PMC8288016

[B60] Hirochika H. Sakaguchi K. (1982). Analysis of linear plasmids isolated from *Streptomyces*: association of protein with the ends of the plasmid DNA. *Plasmid* 7 59–65. 10.1016/0147-619X(82)90027-0 6283574

[B61] Jha J. K. Demarre G. Venkova-Canova T. Chattoraj D. K. (2012). Replication regulation of *Vibrio cholerae* chromosome II involves initiator binding to the origin both as monomer and as dimer. *Nucleic Acids Res.* 40 6026–6038. 10.1093/nar/gks260 22447451 PMC3401445

[B62] Jha J. K. Ghirlando R. Chattoraj D. K. (2014). Initiator protein dimerization plays a key role in replication control of *Vibrio cholerae* chromosome 2. *Nucleic Acids Res.* 42 10538–10549. 10.1093/nar/gku771 25159619 PMC4176361

[B63] Jha J. K. Li M. Ghirlando R. Miller Jenkins L. M. Wlodawer A. Chattoraj D. (2017). The DnaK chaperone uses different mechanisms to promote and inhibit replication of *Vibrio cholerae* chromosome 2. *mBio* 8:e000427-17. 10.1128/mBio.00427-17 28420739 PMC5395669

[B64] Jumas-Bilak E. Michaux-Charachon S. Bourg G. O’Callaghan D. Ramuz M. (1998). Differences in chromosome number and genome rearrangements in the genus *Brucella*. *Mol. Microbiol.* 27 99–106. 10.1046/j.1365-2958.1998.00661.x 9466259

[B65] Kadoya R. Baek J. H. Sarker A. Chattoraj D. K. (2011). Participation of chromosome segregation protein ParAI of *Vibrio cholerae* in chromosome replication. *J. Bacteriol.* 193 1504–1514. 10.1128/JB.01067-10 21257772 PMC3067663

[B66] Kahng L. S. Shapiro L. (2001). The CcrM DNA methyltransferase of *Agrobacterium tumefaciens* is essential, and its activity is cell cycle regulated. *J. Bacteriol.* 183 3065–3075. 10.1128/JB.183.10.3065-3075.2001 11325934 PMC95206

[B67] Kahng L. S. Shapiro L. (2003). Polar localization of replicon origins in the multipartite genomes of *Agrobacterium tumefaciens* and *Sinorhizobium meliloti*. *J. Bacteriol.* 185 3384–3391. 10.1128/JB.185.11.3384-3391.2003 12754237 PMC155372

[B68] Kasho K. Katayama T. (2013). DnaA binding locus datA promotes DnaA-ATP hydrolysis to enable cell cycle-coordinated replication initiation. *Proc. Natl. Acad. Scci. U.S.A.* 110 936–941. 10.1073/pnas.1212070110 23277577 PMC3549119

[B69] Katayama T. Ozaki S. Keyamura K. Fujimitsu K. (2010). Regulation of the replication cycle: conserved and diverse regulatory systems for DnaA and oriC. *Nat. Rev. Microbiol.* 8 163–170. 10.1038/nrmicro2314 20157337

[B70] Kawakami H. Keyamura K. Katayama T. (2005). Formation of an ATP-DnaA-specific initiation complex requires DnaA Arginine 285, a conserved motif in the AAA+ protein family. *J. Biol. Chem.* 280 27420–27430. 10.1074/jbc.M502764200 15901724

[B71] Kemter F. S. Messerschmidt S. J. Schallopp N. Sobetzko P. Lang E. Bunk B. (2018). Synchronous termination of replication of the two chromosomes is an evolutionary selected feature in Vibrionaceae. *PLoS Genet.* 14:e1007251. 10.1371/journal.pgen.1007251 29505558 PMC5854411

[B72] Kitagawa R. Ozaki T. Moriya S. Ogawa T. (1998). Negative control of replication initiation by a novel chromosomal locus exhibiting exceptional affinity for *Escherichia coli* DnaA protein. *Genes Dev.* 12 3032–3043. 10.1101/gad.12.19.3032 9765205 PMC317192

[B73] Koch B. Ma X. Løbner-Olesen A. (2010). Replication of *Vibrio cholerae* chromosome I in *Escherichia coli*: dependence on dam methylation. *J. Bacteriol.* 192 3903–3914. 10.1128/JB.00311-10 20511501 PMC2916376

[B74] Koch B. Ma X. Løbner-Olesen A. (2012). rctB mutations that increase copy number of *Vibrio cholerae* oriCII in *Escherichia coli*. *Plasmid* 68 159–169. 10.1016/j.plasmid.2012.03.003 22487081

[B75] Komori H. Matsunaga F. Higuchi Y. Ishiai M. Wada C. Miki K. (1999). Crystal structure of a prokaryotic replication initiator protein bound to DNA at 2.6 A resolution. *EMBO J.* 18 4597–4607. 10.1093/emboj/18.17.4597 10469640 PMC1171534

[B76] Koper P. Żebracki K. Marczak M. Skorupska A. Mazur A. (2016). RepB proteins of the multipartite *Rhizobium leguminosarum* bv. *trifolii* genome discriminate between centromere-like parS sequences for plasmid segregational stability. *Mol. Microbiol.* 102 446–466. 10.1111/mmi.13472 27480612

[B77] Landeta C. Dávalos A. Cevallos M. Á. Geiger O. Brom S. Romero D. (2011). Plasmids with a chromosome-like role in rhizobia. *J. Bacteriol.* 193 1317–1326. 10.1128/JB.01184-10 21217003 PMC3067620

[B78] Lesic B. Zouine M. Ducos-Galand M. Huon C. Rosso M.-L. Prévost M.-C. (2012). A natural system of chromosome transfer in *Yersinia pseudotuberculosis*. *PLoS Genet.* 8:e1002529. 10.1371/journal.pgen.1002529 22412380 PMC3297565

[B79] Lu Y. B. Datta H. J. Bastia D. (1998). Mechanistic studies of initiator-initiator interaction and replication initiation. *EMBO J.* 17 5192–5200. 10.1093/emboj/17.17.5192 9724655 PMC1170847

[B80] MacLean A. M. Finan T. M. Sadowsky M. J. (2007). Genomes of the symbiotic nitrogen-fixing bacteria of legumes. *Plant Physiol.* 144 615–622. 10.1104/pp.107.101634 17556525 PMC1914180

[B81] MacLellan S. R. Smallbone L. A. Sibley C. D. Finan T. M. (2005). The expression of a novel antisense gene mediates incompatibility within the large *repABC* family of alpha-proteobacterial plasmids. *Mol. Microbiol.* 55 611–623. 10.1111/j.1365-2958.2004.04412.x 15659174

[B82] MacLellan S. R. Zaheer R. Sartor A. L. MacLean A. M. Finan T. M. (2006). Identification of a megaplasmid centromere reveals genetic structural diversity within the repABC family of basic replicons. *Mol. Microbiol.* 59 1559–1575. 10.1111/j.1365-2958.2006.05040.x 16468995

[B83] Marchetti M. Capela D. Glew M. Cruveiller S. Chane-Woon-Ming B. Gris C. (2010). Experimental evolution of a plant pathogen into a legume symbiont. *PLoS Biol.* 8:e1000280. 10.1371/journal.pbio.1000280 20084095 PMC2796954

[B84] Marczynski G. T. (1999). Chromosome methylation and measurement of faithful, once and only once per cell cycle chromosome replication in *Caulobacter crescentus*. *J. Bacteriol.* 181 1984–1993. 10094673 10.1128/jb.181.7.1984-1993.1999 PMC93608

[B85] Marczynski G. T. Shapiro L. (2002). Control of chromosome replication in *Caulobacter crescentus*. *Annu. Rev. Microbiol.* 56 625–656. 10.1146/annurev.micro.56.012302.161103 12142494

[B86] Messerschmidt S. J. Kemter F. S. Schindler D. Waldminghaus T. (2015). Synthetic secondary chromosomes in *Escherichia coli* based on the replication origin of chromosome II in *Vibrio cholerae*. *Biotechnol. J.* 10 302–314. 10.1002/biot.201400031 25359671

[B87] Michaux S. Paillisson J. Carles-Nurit M. J. Bourg G. Allardet-Servent A. Ramuz M. (1993). Presence of two independent chromosomes in the *Brucella melitensis* 16M genome. *J. Bacteriol.* 175 701–705. 10.1128/jb.175.3.701-705.1993 8423146 PMC196208

[B88] Mohapatra S. S. Fioravanti A. Biondi E. G. (2014). DNA methylation in *Caulobacter* and other *Alphaproteobacteria* during cell cycle progression. *Trends Microbiol.* 22 528–535. 10.1016/j.tim.2014.05.003 24894626

[B89] Moreno E. (1998). Genome evolution within the alpha *Proteobacteria*: why do some bacteria not possess plasmids and others exhibit more than one different chromosome? *FEMS Microbiol. Rev.* 22 255–275. 10.1111/j.1574-6976.1998.tb00370.x 9862123

[B90] Murray H. Errington J. (2008). Dynamic control of the DNA replication initiation protein DnaA by Soj/ParA. *Cell* 135 74–84. 10.1016/j.cell.2008.07.044 18854156

[B91] Nakamura A. Wada C. Miki K. (2007). Structural basis for regulation of bifunctional roles in replication initiator protein. *Proc. Natl. Acad. Sci. U.S.A.* 104 18484–18489. 10.1073/pnas.0705623104 18000058 PMC2141803

[B92] Norais C. Hawkins M. Hartman A. L. Eisen J. A. Myllykallio H. Allers T. (2007). Genetic and physical mapping of DNA replication origins in *Haloferax volcanii*. *PLoS Genet.* 3:e00077. 10.1371/journal.pgen.0030077 17511521 PMC1868953

[B93] Nordström K. Dasgupta S. (2006). Copy-number control of the *Escherichia coli* chromosome: a plasmidologist’s view. *EMBO Rep.* 7 484–489. 10.1038/sj.embor.7400681 16670681 PMC1479556

[B94] Ogura Y. Ogasawara N. Harry E. J. Moriya S. (2003). Increasing the ratio of Soj to Spo0J promotes replication initiation in *Bacillus subtilis*. *J. Bacteriol.* 185 6316–6324. 10.1128/JB.185.21.6316-6324.2003 14563866 PMC219394

[B95] Ohbayashi R. Watanabe S. Ehira S. Kanesaki Y. Chibazakura T. Yoshikawa H. (2016). Diversification of DnaA dependency for DNA replication in cyanobacterial evolution. *ISME J.* 10 1113–1121. 10.1038/ismej.2015.194 26517699 PMC5029214

[B96] Okada K. Iida T. Kita-Tsukamoto K. Honda T. (2005). Vibrios commonly possess two chromosomes. *J. Bacteriol.* 187 752–757. 10.1128/JB.187.2.752-757.2005 15629946 PMC543535

[B97] Orlova N. Gerding M. Ivashkiv O. Olinares P. D. B. Chait B. T. Waldor M. K. (2017). The replication initiator of the cholera pathogen’s second chromosome shows structural similarity to plasmid initiators. *Nucleic Acids Res.* 45 3724–3737. 10.1093/nar/gkw1288 28031373 PMC5397143

[B98] Pal D. Venkova-Canova T. Srivastava P. Chattoraj D. K. (2005). Multipartite regulation of rctB, the replication initiator gene of *Vibrio cholerae* chromosome II. *J. Bacteriol.* 187 7167–7175. 10.1128/JB.187.21.7167-7175.2005 16237000 PMC1272990

[B99] Palmer K. M. Turner S. L. Young J. P. (2000). Sequence diversity of the plasmid replication gene repC in the Rhizobiaceae. *Plasmid* 44 209–219. 10.1006/plas.2000.1488 11078647

[B100] Pappas K. M. Winans S. C. (2003a). A LuxR-type regulator from *Agrobacterium tumefaciens* elevates Ti plasmid copy number by activating transcription of plasmid replication genes. *Mol. Microbiol.* 48 1059–1073. 12753196 10.1046/j.1365-2958.2003.03488.x

[B101] Pappas K. M. Winans S. C. (2003b). The RepA and RepB autorepressors and TraR play opposing roles in the regulation of a Ti plasmid repABC operon. *Mol. Microbiol.* 49 441–455. 12828641 10.1046/j.1365-2958.2003.03560.x

[B102] Pastorino G. N. Martinez Alcántara V. Balatti P. A. (2003). Identification of fast and slow growing rhizobia nodulating soybean (*Glycine max* [L.] Merr) by a multiplex PCR reaction. *FEMS Microbiol. Lett.* 229 153–158. 10.1016/S0378-1097(03)00796-1 14680692

[B103] Petersen J. Brinkmann H. Pradella S. (2009). Diversity and evolution of *repABC* type plasmids in *Rhodobacterales*. *Environ. Microbiol.* 11 2627–2638. 10.1111/j.1462-2920.2009.01987.x 19601964

[B104] Petersen J. Frank O. Göker M. Pradella S. (2013). Extrachromosomal, extraordinary and essential–the plasmids of the *Roseobacter* clade. *Appl. Microbiol. Biotechnol.* 97 2805–2815. 10.1007/s00253-013-4746-8 23435940

[B105] Pini F. De Nisco N. J. Ferri L. Penterman J. Fioravanti A. Brilli M. (2015). Cell cycle control by the master regulator CtrA in *Sinorhizobium meliloti*. *PLoS Genet.* 11:e1005232. 10.1371/journal.pgen.1005232 25978424 PMC4433202

[B106] Pinto U. M. Flores-Mireles A. L. Costa E. D. Winans S. C. (2011). RepC protein of the octopine-type Ti plasmid binds to the probable origin of replication within repC and functions only in cis. *Mol. Microbiol.* 81 1593–1606. 10.1111/j.1365-2958.2011.07789.x 21883520

[B107] Pinto U. M. Pappas K. M. Winans S. C. (2012). The ABCs of plasmid replication and segregation. *Nat. Rev. Microbiol.* 10 755–765. 10.1038/nrmicro2882 23070556

[B108] Pritchard R. H. Chandler M. G. Collins J. (1975). Independence of F replication and chromosome replication in *Escherichia coli*. *Mol. Gen. Genet.* 138 143–155. 10.1007/BF02428118 1105149

[B109] Prozorov A. A. (2008). Additional chromosomes in bacteria: properties and origin. *Mikrobiologiia* 77 437–447. 10.1134/S0026261708040012 18825968

[B110] Quon K. C. Marczynski G. T. Shapiro L. (1996). Cell cycle control by an essential bacterial two-component signal transduction protein. *Cell* 84 83–93. 10.1016/S0092-8674(00)80995-2 8548829

[B111] Rajewska M. Wegrzyn K. Konieczny I. (2012). AT-rich region and repeated sequences - the essential elements of replication origins of bacterial replicons. *FEMS Microbiol. Rev.* 36 408–434. 10.1111/j.1574-6976.2011.00300.x 22092310

[B112] Ramachandran R. Ciacca P. N. Filsuf T. A. Jha J. K. Chattoraj D. K. (2018). Chromosome 1 licenses chromosome 2 replication in *Vibrio cholerae* by doubling the crtS gene dosage. *PLoS Genet.* 14:e1007426. 10.1371/journal.pgen.1007426 29795553 PMC5991422

[B113] Ramírez-Romero M. A. Soberón N. Pérez-Oseguera A. Téllez-Sosa J. Cevallos M. A. (2000). Structural elements required for replication and incompatibility of the *Rhizobium etli* symbiotic plasmid. *J. Bacteriol.* 182 3117–3124. 10.1128/JB.182.11.3117-3124.2000 10809690 PMC94497

[B114] Ramírez-Romero M. A. Téllez-Sosa J. Barrios H. Pérez-Oseguera A. Rosas V. Cevallos M. A. (2001). RepA negatively autoregulates the transcription of the repABC operon of the *Rhizobium etli* symbiotic plasmid basic replicon. *Mol. Microbiol.* 42 195–204. 10.1046/j.1365-2958.2001.02621.x 11679078

[B115] Rasmussen T. Jensen R. B. Skovgaard O. (2007). The two chromosomes of *Vibrio cholerae* are initiated at different time points in the cell cycle. *EMBO J.* 26 3124–3131. 10.1038/sj.emboj.7601747 17557077 PMC1914095

[B116] Reyes-Lamothe R. Nicolas E. Sherratt D. J. (2012). Chromosome replication and segregation in bacteria. *Annu. Rev. Genet.* 46 121–143. 10.1146/annurev-genet-110711-155421 22934648

[B117] Robertson G. T. Reisenauer A. Wright R. Jensen R. B. Jensen A. Shapiro L. (2000). The *Brucella abortus* CcrM DNA methyltransferase is essential for viability, and its overexpression attenuates intracellular replication in murine macrophages. *J. Bacteriol.* 182 3482–3489. 10.1128/JB.182.12.3482-3489.2000 10852881 PMC101938

[B118] Rosenberg C. Casse-Delbart F. Dusha I. David M. Boucher C. (1982). Megaplasmids in the plant-associated bacteria *Rhizobium meliloti* and *Pseudomonas solanacearum*. *J. Bacteriol.* 150 402–406. 7061401 10.1128/jb.150.1.402-406.1982 PMC220129

[B119] Schallopp N. Milbredt S. Sperlea T. Kemter F. S. Bruhn M. Schindler D. (2017). Establishing a system for testing replication inhibition of the *Vibrio cholerae* secondary chromosome in *Escherichia coli*. *Antibiotics* 7:E3. 10.3390/antibiotics7010003 29295515 PMC5872114

[B120] Schekman R. Weiner A. Kornberg A. (1974). Multienzyme systems of DNA replication. *Science* 186 987–993. 10.1126/science.186.4168.987 4620044

[B121] Scholefield G. Errington J. Murray H. (2012). Soj/ParA stalls DNA replication by inhibiting helix formation of the initiator protein DnaA. *EMBO J.* 31 1542–1555. 10.1038/emboj.2012.6 22286949 PMC3321191

[B122] Silva A. J. Benitez J. A. (2016). *Vibrio cholerae* biofilms and cholera pathogenesis. *PLoS Negl. Trop. Dis.* 10:e0004330. 10.1371/journal.pntd.0004330 26845681 PMC4741415

[B123] Soberón N. Venkova-Canova T. Ramírez-Romero M. A. Téllez-Sosa J. Cevallos M. A. (2004). Incompatibility and the partitioning site of the repABC basic replicon of the symbiotic plasmid from *Rhizobium etli*. *Plasmid* 51 203–216. 10.1016/j.plasmid.2004.01.005 15109827

[B124] Soora M. Tomasch J. Wang H. Michael V. Petersen J. Engelen B. (2015). Oxidative stress and starvation in *Dinoroseobacter shibae*: the role of extrachromosomal elements. *Front. Microbiol.* 6:233. 10.3389/fmicb.2015.00233 25859246 PMC4373377

[B125] Stephens C. Reisenauer A. Wright R. Shapiro L. (1996). A cell cycle-regulated bacterial DNA methyltransferase is essential for viability. *Proc. Natl. Acad. Sci. U.S.A.* 93 1210–1214. 10.1073/pnas.93.3.1210 8577742 PMC40058

[B126] Suwanto A. Kaplan S. (1989). Physical and genetic mapping of the *Rhodobacter sphaeroides* 2.4.1 genome: presence of two unique circular chromosomes. *J. Bacteriol.* 171 5850–5859. 10.1128/jb.171.11.5850-5859.1989 2808300 PMC210445

[B127] Swan M. K. Bastia D. Davies C. (2006). Crystal structure of pi initiator protein-iteron complex of plasmid R6K: implications for initiation of plasmid DNA replication. *Proc. Natl. Acad. Sci. U.S.A.* 103 18481–18486. 10.1073/pnas.0609046103 17124167 PMC1693688

[B128] Szymanik M. Welc-Faleciak R. Bartosik D. Włodarczyk M. (2006). Replication system of plasmid pMTH4 of *Paracoccus methylutens* DM12 contains an enhancer. *Pol. J. Microbiol.* 55 261–270. 17416062

[B129] Touchon M. Rocha E. P. C. (2016). Coevolution of the organization and structure of prokaryotic genomes. *Cold Spring Harb. Perspect. Biol.* 8:a018168. 10.1101/cshperspect.a018168 26729648 PMC4691797

[B130] Trucksis M. Michalski J. Deng Y. K. Kaper J. B. (1998). The *Vibrio cholerae* genome contains two unique circular chromosomes. *Proc. Natl. Acad. Sci. U.S.A.* 95 14464–14469. 10.1073/pnas.95.24.14464 9826723 PMC24396

[B131] Val M.-E. Kennedy S. P. El Karoui M. Bonné L. Chevalier F. Barre F.-X. (2008). FtsK-dependent dimer resolution on multiple chromosomes in the pathogen *Vibrio cholerae*. *PLoS Genet.* 4:e1000201. 10.1371/journal.pgen.1000201 18818731 PMC2533119

[B132] Val M.-E. Kennedy S. P. Soler-Bistué A. J. Barbe V. Bouchier C. Ducos-Galand M. (2014). Fuse or die: how to survive the loss of Dam in *Vibrio cholerae*. *Mol. Microbiol.* 91 665–678. 10.1111/mmi.12483 24308271

[B133] Val M.-E. Marbouty M. de Lemos Martins F. Kennedy S. P. Kemble H. Bland M. J. (2016). A checkpoint control orchestrates the replication of the two chromosomes of *Vibrio cholerae*. *Sci. Adv.* 2:e1501914. 10.1126/sciadv.1501914 27152358 PMC4846446

[B134] Val M.-E. Skovgaard O. Ducos-Galand M. Bland M. J. Mazel D. (2012). Genome engineering in *Vibrio cholerae*: a feasible approach to address biological issues. *PLoS Genet.* 8:e1002472. 10.1371/journal.pgen.1002472 22253612 PMC3257285

[B135] Venkova-Canova T. Baek J. H. Fitzgerald P. C. Blokesch M. Chattoraj D. K. (2013). Evidence for two different regulatory mechanisms linking replication and segregation of *Vibrio cholerae* chromosome II. *PLoS Genet.* 9:e1003579. 10.1371/journal.pgen.1003579 23818869 PMC3688505

[B136] Venkova-Canova T. Chattoraj D. K. (2011). Transition from a plasmid to a chromosomal mode of replication entails additional regulators. *Proc. Natl. Acad. Sci. U.S.A.* 108 6199–6204. 10.1073/pnas.1013244108 21444815 PMC3076835

[B137] Venkova-Canova T. Saha A. Chattoraj D. K. (2012). A 29-mer site regulates transcription of the initiator gene as well as function of the replication origin of *Vibrio cholerae* chromosome II. *Plasmid* 67 102–110. 10.1016/j.plasmid.2011.12.009 22248922 PMC3319240

[B138] Venkova-Canova T. Soberón N. E. Ramírez-Romero M. A. Cevallos M. A. (2004). Two discrete elements are required for the replication of a repABC plasmid: an antisense RNA and a stem-loop structure. *Mol. Microbiol.* 54 1431–1444. 10.1111/j.1365-2958.2004.04366.x 15554980

[B139] Venkova-Canova T. Srivastava P. Chattoraj D. K. (2006). Transcriptional inactivation of a regulatory site for replication of *Vibrio cholerae* chromosome II. *Proc. Natl. Acad. Sci. U.S.A.* 103 12051–12056. 10.1073/pnas.0605120103 16873545 PMC1567695

[B140] Wegrzyn K. E. Gross M. Uciechowska U. Konieczny I. (2016). Replisome assembly at bacterial chromosomes and iteron plasmids. *Front. Mol. Biosci.* 3:39. 10.3389/fmolb.2016.00039 27563644 PMC4980987

[B141] Wickner S. Hoskins J. McKenney K. (1991). Monomerization of RepA dimers by heat shock proteins activates binding to DNA replication origin. *Proc. Natl. Acad. Sci. U.S.A.* 88 7903–7907. 10.1073/pnas.88.18.7903 1896443 PMC52413

[B142] Williams D. R. Thomas C. M. (1992). Active partitioning of bacterial plasmids. *J. Gen. Microbiol.* 138 1–16. 10.1099/00221287-138-1-1 1556544

[B143] Wion D. Casadesús J. (2006). N6-methyl-adenine: an epigenetic signal for DNA-protein interactions. *Nat. Rev. Microbiol.* 4 183–192. 10.1038/nrmicro1350 16489347 PMC2755769

[B144] Wolański M. Jakimowicz D. Zakrzewska-Czerwińska J. (2014). Fifty years after the replicon hypothesis: cell-specific master regulators as new players in chromosome replication control. *J. Bacteriol.* 196 2901–2911. 10.1128/JB.01706-14 24914187 PMC4135643

[B145] Wright R. Stephens C. Shapiro L. (1997). The CcrM DNA methyltransferase is widespread in the alpha subdivision of *proteobacteria*, and its essential functions are conserved in *Rhizobium meliloti* and *Caulobacter crescentus*. *J. Bacteriol.* 179 5869–5877. 10.1128/jb.179.18.5869-5877.1997 9294447 PMC179479

[B146] Xu Q. Dziejman M. Mekalanos J. J. (2003). Determination of the transcriptome of *Vibrio cholerae* during intraintestinal growth and midexponential phase *in vitro*. *Proc. Natl. Acad. Sci. U.S.A.* 100 1286–1291. 10.1073/pnas.0337479100 12552086 PMC298765

[B147] Yamaichi Y. Fogel M. A. McLeod S. M. Hui M. P. Waldor M. K. (2007). Distinct centromere-like parS sites on the two chromosomes of *Vibrio* spp. *J. Bacteriol.* 189 5314–5324. 10.1128/JB.00416-07 17496089 PMC1951861

[B148] Yamaichi Y. Gerding M. A. Davis B. M. Waldor M. K. (2011). Regulatory cross-talk links *Vibrio cholerae* chromosome II replication and segregation. *PLoS Genet.* 7:e1002189. 10.1371/journal.pgen.1002189 21811418 PMC3141006

[B149] Yamaichi Y. Iida T. Park K. S. Yamamoto K. Honda T. (1999). Physical and genetic map of the genome of *Vibrio parahaemolyticus*: presence of two chromosomes in *Vibrio* species. *Mol. Microbiol.* 31 1513–1521. 10.1046/j.1365-2958.1999.01296.x 10200969

[B150] Yao Y. Enkhtsetseg S. Odsbu I. Fan L. Morigen M. (2018). Mutations of DnaA-boxes in the oriR region increase replication frequency of the MiniR1-1 plasmid. *BMC Microbiol.* 18:27. 10.1186/s12866-018-1162-3 29614952 PMC5883639

[B151] Żebracki K. Koper P. Marczak M. Skorupska A. Mazur A. (2015). Plasmid-encoded RepA proteins specifically autorepress individual repABC operons in the multipartite *Rhizobium leguminosarum* bv. *trifolii* Genome. *PLoS One* 10:e0131907. 10.1371/journal.pone.0131907 26147968 PMC4492784

[B152] Zeuthen J. Pato M. L. (1971). Replication of the F’lac sex factor in the cell cycle of *Escherichia coli*. *Mol. Gen. Genet.* 111 242–255. 10.1007/BF00433109 4935354

[B153] Zhong Z. Helinski D. Toukdarian A. (2003). A specific region in the N terminus of a replication initiation protein of plasmid RK2 is required for recruitment of *Pseudomonas aeruginosa* DnaB helicase to the plasmid origin. *J. Biol. Chem.* 278 45305–45310. 10.1074/jbc.M306058200 12952979

[B154] Zuerner R. L. Herrmann J. L. Saint Girons I. (1993). Comparison of genetic maps for two *Leptospira interrogans* serovars provides evidence for two chromosomes and intraspecies heterogeneity. *J. Bacteriol.* 175 5445–5451. 10.1128/jb.175.17.5445-5451.1993 7690025 PMC206600

